# Complex Cooperative Functions of Heparan Sulfate Proteoglycans Shape Nervous System Development in *Caenorhabditis elegans*

**DOI:** 10.1534/g3.114.012591

**Published:** 2014-08-05

**Authors:** Carlos A. Díaz-Balzac, María I. Lázaro-Peña, Eillen Tecle, Nathali Gomez, Hannes E. Bülow

**Affiliations:** * Department of Genetics, Albert Einstein College of Medicine, Bronx, New York, 10461; † Dominick P. Purpura Department of Neuroscience, Albert Einstein College of Medicine, Bronx, New York, 10461

**Keywords:** *C. elegans*, Kallmann syndrome, development, heparan, nervous system

## Abstract

The development of the nervous system is a complex process requiring the integration of numerous molecular cues to form functional circuits. Many cues are regulated by heparan sulfates, a class of linear glycosaminoglycan polysaccharides. These sugars contain distinct modification patterns that regulate protein–protein interactions. Misexpressing the homolog of KAL-1/anosmin-1, a neural cell adhesion molecule mutant in Kallmann syndrome, in *Caenorhabditis elegans* causes a highly penetrant, heparan sulfate–dependent axonal branching phenotype in AIY interneurons. In an extended forward genetic screen for modifiers of this phenotype, we identified alleles in new as well as previously identified genes involved in HS biosynthesis and modification, namely the xylosyltransferase *sqv-6*, the HS-6-*O*-sulfotransferase *hst-6*, and the HS-3-*O*-sulfotransferase *hst-3.2*. Cell-specific rescue experiments showed that different HS biosynthetic and modification enzymes can be provided cell-nonautonomously by different tissues to allow *kal-1*-dependent branching of AIY. In addition, we show that heparan sulfate proteoglycan core proteins that carry the heparan sulfate chains act genetically in a highly redundant fashion to mediate *kal-1*-dependent branching in AIY neurons. Specifically, *lon-2*/glypican and *unc-52*/perlecan act in parallel genetic pathways and display synergistic interactions with *sdn-1*/syndecan to mediate *kal-1* function. Because all of these heparan sulfate core proteins have been shown to act in different tissues, these studies indicate that KAL-1/anosmin-1 requires heparan sulfate with distinct modification patterns of different cellular origin for function. Our results support a model in which a three-dimensional scaffold of heparan sulfate mediates KAL-1/anosmin-1 and intercellular communication through complex and cooperative interactions. In addition, the genes we have identified could contribute to the etiology of Kallmann syndrome in humans.

The extracellular matrix (ECM) provides a scaffold for the development and function of tissues and organs. For example, the nervous system makes use of the wide range of signals found in the ECM to mediate processes such as cell migration, axon guidance, and neurite branching (Porcionatto 2006; Zimmermann and Dours-Zimmermann 2008; Myers *et al.* 2011). Heparan sulfate proteoglycans (HSPGs) are key components of the ECM in mediating nervous system development (Yamaguchi 2001; Bülow and Hobert 2006; Van Vactor *et al.* 2006). HSPGs exist in membrane-bound forms, such as syndecans and glypicans, or are secreted, such as perlecan, agrin, or collagen XVIII (Figure 1A) (Bernfield *et al.* 1999). Many but not all functions of HSPGs are mediated by the heparan sulfate (HS) chains attached to their extracellular domain (Häcker *et al.* 1997). These HS are linear glycosaminoglycan polysaccharides consisting of a characteristic disaccharide repeat of hexuronic acid with glucosamine that can be heavily and diversely modified (Figure 1B). The modifications include sulfations, epimerization, and acetylation of different sugar moieties and are introduced by specific enzymes in the Golgi (Figure 1, B and C) (Lindahl *et al.* 1998; Esko and Selleck 2002; Lindahl and Li 2009). HS chains are known to function as co-factors and have previously been shown to be part of many signaling pathways (Bülow and Hobert 2006; Bishop *et al.* 2007), including but not limited to the fibroblast growth factor receptor FGFR, the Slit/Robo ligand/receptor cassette of axon guidance factors, and also the neural cell adhesion molecule KAL1/anosmin-1, which causes hereditary Kallmann syndrome (KS)/idiopathic hypogonadotropic hypogonadism (IHH) (Franco *et al.* 1991; Legouis *et al.* 1991).

**Figure 1:**
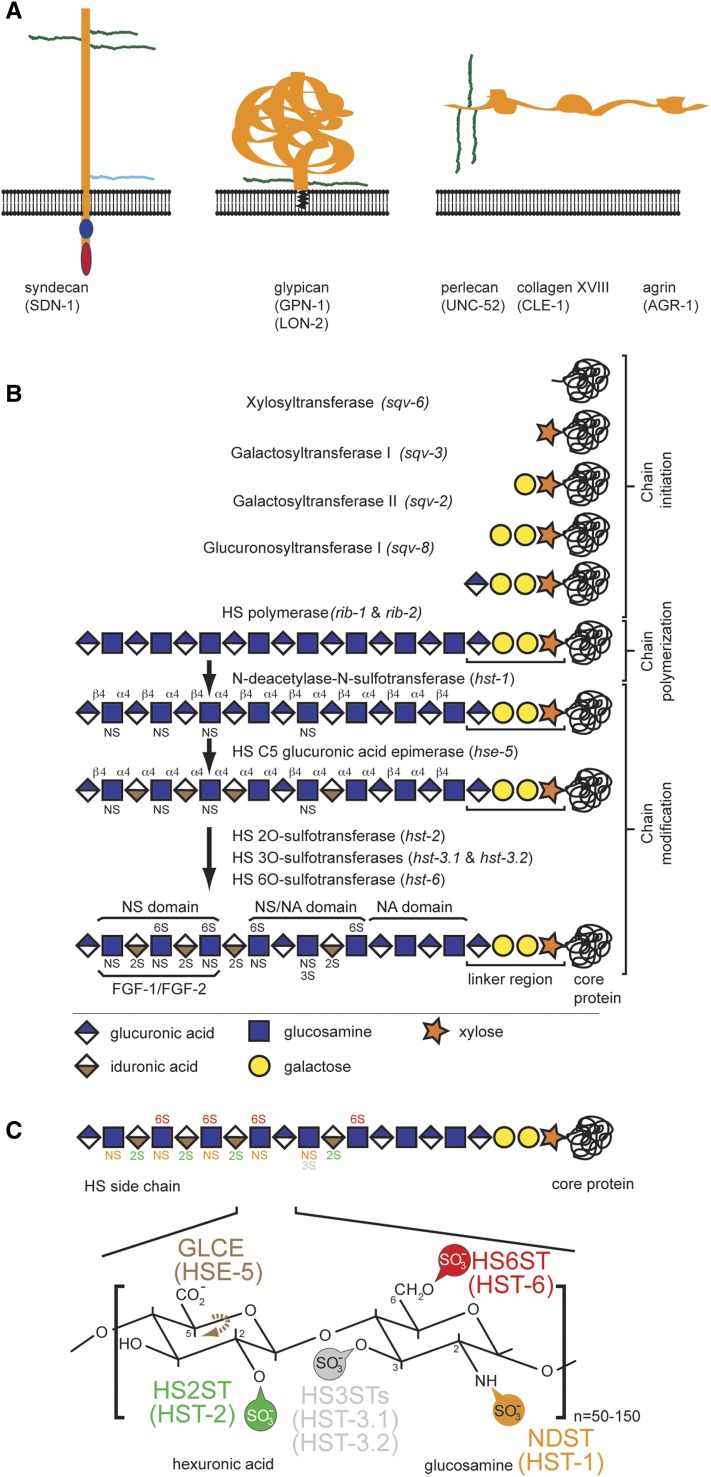
Schematics of heparan sulfate proteoglycans (HSPGs) and heparan sulfate glycosaminoglycans. (A) Heparan sulfate proteoglycans (HSPG) are found in either membrane-bound forms, such as syndecans (*sdn-1*) and glypicans (*lon-2* and *gpn-1*), or secreted forms, such as perlecan (*unc-52*), agrin (*agr-1*), and collagen XVIII (*cle-1*). *C. elegans* proteins/genes in all panels are shown in parentheses. HS chains are indicated in green and chondroitin sulfate chains are shown in light blue. (B) Schematic of heparan sulfate chain biosynthesis [adapted from Esko and Lindahl (2001)], which is initiated by a series of reactions that add an invariable tetrasaccharide linker to a serine of a core protein (Esko and Zhang 1996), followed by elongation through the addition of disaccharide repeats (C). Concomitantly, the disaccharides are modified nonuniformly by modifying enzymes, thereby creating unique motifs. (C) Schematic of the characteristic disaccharide repeat. Relevant modification enzymes (with *C. elegans* gene names in parentheses) and the positions they modify are indicated: NDST, (*N*-decacetylase-sulfotransferase); GLCE, (*C5*-glucuronyl-epimerase); HS2ST, HS-2-*O*- sulfotransferase; HS3STs, HS-3-*O*-sulfotransferases; HS6ST, HS-6-*O*-sulfotransferase.

KS/IHH is a heterogeneous syndrome characterized by lack of sexual maturation and infertility (Seminara *et al.* 1998). IHH patients may exhibit a normal sense of smell [normosmic IHH (nIHH)] or a lack of sense of smell (anosmia). Historically, the association of IHH with anosmia has been termed KS. KS is considered a neuronal targeting defect because the olfactory axons fail to reach their targets in the olfactory bulb and GnRH-secreting neurons fail to migrate to the hypothalamus (Lutz *et al.* 1993). To date, 21 genes associated with KS/nIHH have been identified, namely, KAL1, FGFR1, FGF8, PROKR2, PROK2, CHD7, FGF8, GNRHR, KISS1R, NELF, TAC, TAC3R, GNRH1, KISS1, WDR11, HS6ST1, SEMA3A, SPRY4, IL17RD, DUSP6, FGF17, and FLRT3. Even though a considerable number of disease-causing genes are known, these only account for approximately 30% of the cases of patients with KS/nIHH (Dodé *et al.* 2006; Hardelin and Dodé 2008). Therefore, a substantial number of genes remain to be identified as being associated with the pathophysiology of KS/nIHH.

Mechanistic studies of the pathophysiology of KS/nIHH and specifically the neural cell adhesion molecule KAL-1/anosmin-1 have been hampered by the fact that a homolog of KAL1/anosmin-1 cannot be identified in the subfamily of mouse-like rodent genomes, at least not based on primary sequence (Supporting Information, Figure S1). In contrast, sequenced genomes of most, if not all, other vertebrates and several invertebrates, including the small nematode *Caenorhabditis elegans*, encode a KAL1/anosmin-1 homolog (Rugarli and Ballabio 1993; Bülow *et al.* 2002) (Figure S1). Whereas loss of function mutations of *kal-1* result in relatively mild morphogenetic and neuronal phenotypes in worms (Rugarli *et al.* 2002; Hudson *et al.* 2006; Tecle *et al.* 2013), targeted misexpression of *kal-1* in some but not all cellular contexts resulted in strong ectopic neurite branching (Bülow *et al.* 2002). The branching was specific for and dependent on KAL-1. A pilot modifier screen identified several suppressor mutants of *kal-1*-dependent branching, including in the HS-6-*O*-sulfotransferase encoded by *hst-6*, demonstrating that KAL-1 requires HS with specific modifications to exert its branching activity *in vivo* (Bülow *et al.* 2002). Although it was shown that KAL-1 can bind the HSPG core proteins SDN-1/syndecan and GPN-1/glypican in biochemical assays (Hudson *et al.* 2006), the HSPGs mediating neuronal branching remained unknown. The fact that *kal-1* function depended on *hst-6* lead to the prediction that loss of HS6ST1 may have similar phenotypes as loss of *KAL1* in humans; in other words, loss of function mutations in HS6STs could also be found in KS/nIHH patients. Loss of function mutations in the human ortholog of *hst-6*, HS6ST1, were indeed identified in patients with KS/nIHH (Tornberg *et al.* 2011). These findings established that our *C. elegans* approach successfully identified genes involved in KS/nIHH.

To better understand *kal-1* function, we expanded the pilot screen and identified a total of 16 new alleles that modified the *kal-1*-dependent neurite branching, including novel modifiers such as the extracellular protein DIG-1 and the cell adhesion immunoglobulin (Ig) containing molecule SAX-7/L1CAM. We also isolated alleles of *hst-6* and *hst-3.2* (Tecle *et al.* 2013) as well as an allele, *dz147*, which enhanced the branching phenotype and failed to complement a previously identified enhancer mutant (Bülow *et al.* 2002). Genetic analysis of HSPG core protein mutants revealed that HSPG genes act redundantly to mediate *kal-1*-dependent branching. The redundant functions of HSPG are not limited to *kal-1*-dependent branching and are also seen in other cellular contexts, suggesting that this may be a more general theme of HSPG biology. Cell-specific rescue experiments showed that the HS modification enzymes can act cell-nonautonomously during the formation of *kal-1*-dependent branching in AIY, suggesting that *kal-1* requires HS of different cellular origin, each with distinct HS modification patterns for function. Our results support a model of complex cooperative interactions between HS, which may form a three-dimensional scaffold to control activity of signaling molecules.

## Materials and Methods

### *C. elegans* strains and imaging

All strains were maintained using standard methods (Brenner 1974). All experiments were performed at 20° unless otherwise mentioned. All the worms scored were 1-d-old adults unless otherwise specified. Mutant strains used were as follows: LGII: *unc-52(e998)*; LGIII: *hse-5(tm472)*, *dig-1(ky188)*, *dig-1(n1321)*, *dig-1(dz136)*, *dig-1(dz145)*, *dig-1(dz152)*, *dig-1(dz154)*, *dig-1(dz155)*; LGIV: *sax-7(dz156)*; and LGV: *pst-1(ot20)*, *him-5(e1490)*, *ot21*, *dz147*; LGX: *sdn-1(zh20)*, *gpn-1(ok377)*, *lon-2(e678)*, *hst-2(ok595)*, *hst-6(ok273)*, *hst-6(dz134)*, *hst-6(dz151)*, *hst-6(dz168)*, *hst-3.2(tm3006)*, *hst-3.2(dz140)*, *hst-3.2(dz164)*, *hst-3.2(dz169)*, *hst-3.2(dz171)*. Integrated and extrachromosomal arrays were as follows: *mgIs18 [Pttx-3*::*gfp] IV*; *mgIs32 [Pttx-3*::*gfp] III*; *otIs35 [Pttx-3*::*kal-1*; *rol-6(su1006)]X*; *otIs76 [Pttx-3*::*kal-1*; *Punc-122*::*gfp]IV*; *otIs77 [Pttx-3*::*kal-1*; *Punc-122*::*gfp]II*; and *oyIs14 [Psra-6*::*gfp*, *lin-15(+)]V*.

For *dig-1* rescue, rescuing array *rhEx40*, carrying cosmids K07E12 and R05H11, was generated by R. Proenca and E. Hedgecock and was kindly provided by E. Ryder.

### Isolation and molecular identification of mutant alleles

**Figure 2:**
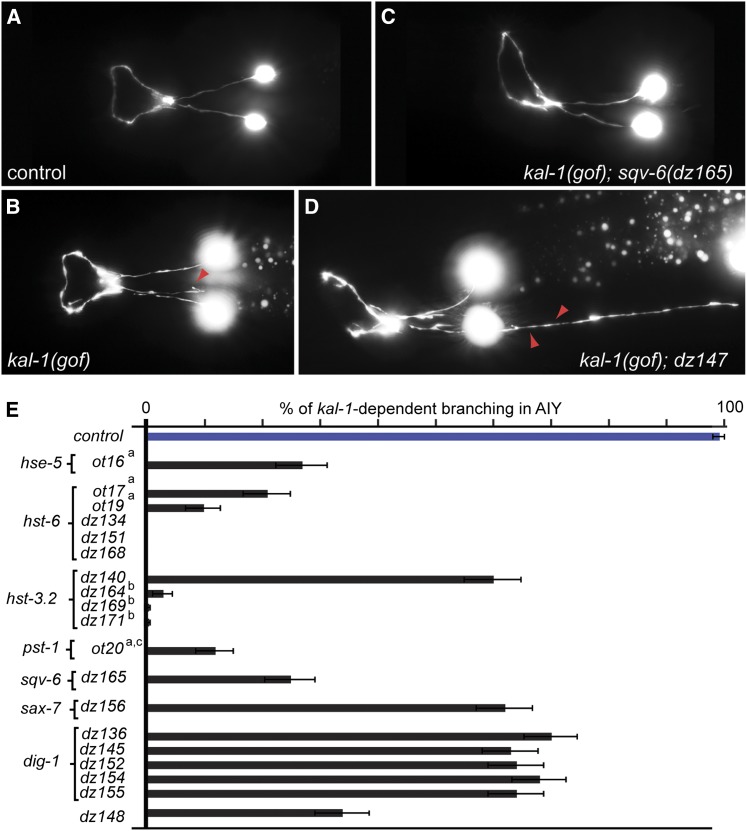
Modifier mutants of the *kal-1*-dependent branching phenotype in AIY. (A–D) Representative images of the *kal-1*-dependent branching in AIY in different mutant backgrounds. Ventral or sublateral views of an adult wild-type animal (*mgIs18 (Is[Pttx-3*::*gfp])*) (A), an animal with *kal-1*-dependent branching in AIY (*otIs76 mgIs18(Is[Pttx-3*::*kal-1*, *Pttx-3*::*gfp]*) (B), a suppressor mutant (*otIs76 mgIs18*; *sqv-6(dz165)*) (C), and an enhancer mutant (*otIs76 mgIs18*; *dz147*) (D). Red arrowheads indicate *kal-1*-dependent branches. Anterior is to the left in all panels. (E) Summary of suppressor mutants identified in the pilot screen described by Bülow *et al.* (2002) and in the extended screen described in this work. The control strain is *otIs35(Is[Pttx-3*::*kal-1])*; *mgIs18(Is[Pttx-3*::*gfp])*, which is fully penetrant for the *kal-1*-dependent branching phenotype. Some of the alleles have been previously described: ^a^Bülow *et al*. (2002), ^b^Tecle *et al.* (2013), ^c^Bhattacharya *et al.* (2009). Error bars indicate the standard error of proportion.

Modifier mutants were isolated from an extension of a pilot F1 clonal screen using ethyl methanesulfonate (EMS) as the mutagen (Bülow *et al.* 2002). A total of 3652 additional haploid genomes were screened. The strain OH125 (*mgIs18*; *otIs35*), which has a 100% penetrant axon branching defect in AIY, was mutagenized with EMS and the F1s were singled-out in individual plates. Four days later, the population of F2s was scored for the suppression/enhancement of the *kal-1*-dependent branches in AIY interneurons. The worms were anesthetized with 10 mM sodium azide and mounted on 5% agarose pads for phenotypic analysis on a Zeiss Axioimager Z1 compound microscope. At least 20 adult animals were scored per plate. Isolated mutants were mapped/cloned by a combination of single nucleotide polymorphisms (SNPs) based approaches (Wicks *et al.* 2001), whole genome sequencing (Doitsidou *et al.* 2010), or by complementation tests with previously identified mutants as indicated below. Three point mutant alleles of the HS-6-*O*-sulfotransferase were fully recessive, showed linkage to the X chromosome, complemented *hst-3.2(tm3006)*, and failed to complement the deletion allele *hst-6(ok273)*. One allele contained a nonsense mutation, resulting in a stop codon after 127 amino acids (*dz134*). Another allele contained a missense mutation (*dz151*), changing a well-conserved negative charge in the 3′ phosphoadenosyl-phophosulfate (PAPS) substrate-binding site to a positive charge (E185K). The third allele introduced a mutation of the splice donor site in exon 1 (Figure 2, Figure S3, Table 2). Three point mutant alleles of the HS-3-*O*-sulfotransferase type II (*hst-3.2*) behaved recessively, displayed X-linkage, but complemented *hst-6(ok273)*. These alleles have been described in detail elsewhere (Tecle *et al.* 2013). A fourth allele, *hst-3.2(dz140)*, contained a mutation in a splice site donor and failed to complement *hst-3.2(dz171)*. This *hst-3.2(dz140)* allele is likely a hypomorphic allele because it only weakly suppressed *kal-1*-dependent branching (Figure 2, Table 2). One allele of the xylosyltransferase *sqv-6* (Hwang *et al.* 2003) displayed linkage to chromosome V and mapping and whole genome sequencing identified the responsible mutation on the left arm of chromosome V (Figure S3) as a mutation in the splice site acceptor of exon 5 (Figure 2, Table 2). This allele, *dz165*, behaved recessively and failed to complement an allele of *pst-1(ot20)* (Bhattacharya *et al.* 2009) but did complement *hse-5(tm472)*. Five alleles of *dig-1*, *dz136*, *dz145*, *dz152*, *dz154*, and *dz155*, behaved recessively and all but *dz136* exhibited an AIY cell body misplacement defect. SNP mapping (Wicks *et al.* 2001) placed *dz152* in the center of chromosome III (data not shown). The *dz152* allele failed to complement an AIY cell body misplacement defect with *dz136*, *dz145*, *dz154*, and *dz155*, and two independently obtained alleles, *dig-1*(*ky188)* and *dig-1(n1321)*. The *dz136* allele was identified by a combination of mapping and whole genomes sequencing and contained a mutation in a splice site. The molecular lesion of the other *dig-1* alleles has not been investigated. One mutant allele of *sax-7* was identified based on linkage to chromosome IV, a penetrant cell positioning defect and noncomplementation with the known *sax-7(nj48)* null allele (Figure 2, Table 2, Table S1). Finally, the *dz148* allele behaved recessively, showed no obvious linkage to chromosome X, IV, or V, and complemented all previously identified mutants. Moreover, no mutation was identified in the coding region of other genes known to be involved in the HS biosynthetic/modification machinery. The enhancer allele *dz147* behaved recessively, showed linkage to chromosome V, and failed to complement the previously identified enhancer *ot21* (Figure 2, Table 2).

### Molecular biology and transgenesis

To assemble tissue-specific expression constructs, the *sqv-6*, *hst-6*, *hse-5*, and *hst-2* cDNAs were cloned under control of the following promoters: hypodermal *dpy-7* (Gilleard *et al.* 1997); body wall muscle *myo-3* (Okkema *et al.* 1993); pan-neuronal *rgef-1* (Altun-Gultekin *et al.* 2001); and an AIY-specific *ttx-3* promoter (Altun-Gultekin *et al.* 2001). All plasmids contained the *unc-54* 3′ UTR and plasmid sequences are available on request. For rescue experiments, the tissue-specific expression constructs were injected into EB2426 [*otIs76mgIs18*; *sqv-6(dz165*)], OH1682 [*otIs76mgIs18*; *hst-6(ok273*)], OH1681 [*hse-5(tm472)*; *otIs76mgIs18*], and OH1945 [*otIs76mgIs18*; *hst-2(ok595*)] at 5 ng/µl together with *Pmyo-3*::*mCherry* as injection marker at 50 ng/µl.

### Statistical analysis

For all proportions, statistical significance was calculated using the z-test, whereas for averages the two-tailed Student *t*-test was used. Statistical significance is indicated throughout the article as follows: ns, not significant; *P < 0.05; **P < 0.005; and ***P < 0.0005.

## Results

### A genetic screen for genes interacting with *kal-1/anosmin-1*

The AIY interneurons are a left/right pair of interneurons that send their axon anteriorly to the nerve ring (White *et al.* 1986). When *kal-1* is overexpressed cell-specifically in AIY, highly penetrant *kal-1*-dependent branching is observed (Figure 2, A and B) (Bülow *et al.* 2002). In an extension of the previously published pilot screen we identified 16 new alleles in a total of 3652 haploid genomes screened that modify the AIY branching phenotype (Figure 2, C–E, Table 1). This brought the number of haploid genomes screened for modification of *kal-1*-dependent branching to a total of 4962 haploid genomes (Table 1). The 16 newly identified mutants fell into six complementation groups of suppressor mutants and one complementation group with an enhancer mutant (Table 1, Table S1). We cloned and identified the molecular lesion in most of the mutant alleles, which are described below (see *Materials and Methods* for details).

**Table 1:** Overview of modifier screens of *kal-1*-dependent branching in AIY interneurons

**Mutagen**	**Genomes Screened**	**Suppressors**	**Enhancers**	**Reference**
EMS	1310	6	1	Bülow *et al.* (2002)
EMS	3652	15	1	This study

The largest complementation group of the expanded screen included five alleles of *dig-1*, which encodes a large extracellular matrix protein (Bénard *et al.* 2006; Burket *et al.* 2006). All alleles suppressed *kal-1*-dependent branching in AIY, behaved recessively, and complemented both each other as well as alleles identified by other laboratories (Table S1). The *dig-1* alleles identified in the screen can be divided into two classes. The first, comprising four alleles (*dz145*, *dz152*, *dz154*, and *dz155*), represented the classical *dig-1* mutant allele, which displays AIY cell positioning defects. Interestingly, the cell positioning defects were already visible during late embryonic stages, but increased with age (Figure S2). These data suggest that DIG-1 also plays a role during neural development in addition to its established role in maintaining cell body position (Bénard *et al.* 2006; Burket *et al.* 2006). The second class comprised a *dig-1* allele that did not exhibit the cell positioning defects, namely *dz136*. All *dig-1* alleles identified in the screen showed suppression of 30% to 40% of *kal-1*-dependent branching in AIY (Figure 2, Table 2). These observations show that branching and cell body positioning are genetically separable, and suggest that *dig-1* function in *kal-1*-dependent branching is not a secondary effect of defects in cell positioning. The *dz136* allele is a splice site acceptor mutation that is predicted to lead to skipping of exon 29 and an in-frame deletion of 145 amino acids in a region of characteristic 70 amino acid repeats (Burket *et al.* 2006). This stretch of amino acids may be important for the function of *dig-1* in *kal-1*-dependent branching, but not for its cell positioning function. We also isolated a point mutant allele (*dz156*) in the immunoglobulin (Ig) containing cell adhesion molecule SAX-7/L1CAM (Figure 2, Table 2) (Salzberg *et al.* 2013) as well as one uncharacterized suppressor (*dz148*) and enhancer allele (*dz147*), respectively (Figure 1, Table 2). Both *dig-1* and *sax-7*/L1CAM have been studied for their functions during maintenance of cell positioning in the nervous system. For instance, both genes have been shown to be required for maintaining the position of cell bodies in the head as well as in the tail of *C. elegans*. Additionally, both also play a role in maintaining the integrity of neural tracts during postembryonic development (Sasakura *et al.* 2005; Bénard *et al.* 2006; Burket *et al.* 2006; Pocock *et al.* 2008). However, these molecules may also play a role during nervous system development. For example, this has been shown for *sax-7*/L1CAM in PVD dendrite morphogenesis (Dong *et al.* 2013; Salzberg *et al.* 2013). In the case of *dig-1*, our experiments provide the first evidence for a developmental role in the nervous system other than its maintenance role (Bénard *et al.* 2006; Burket *et al.* 2006).

**Table 2:** Details of modifier alleles identified in the screen

**Locus**	**Allele**	**Linkage Group**	**Molecular Identity**	**AIY Branching***^a^*	**Pleiotropic Phenotypes**	**Molecular Lesion**
***dig-1***	*dz136*	III	ECM protein	70%		***dig-1***, exon/intron (splice acceptor), *LGIII: 6,777,484 A→T*
	*dz145*			63%	AIY cell body misplacement	Fails to complement *dz152*
	*dz152*			64%	AIY cell body misplacement	Center of LG III by SNP mapping, fails to complement *dig-1(ky188)* and *dig-1(n1321)*
	*dz154*			68%	AIY cell body misplacement	Fails to complement *dz152*
	*dz155*			64%	AIY cell body misplacement	Fails to complement *dz152*
***sax-7***	*dz156*	IV	L1CAM homolog	62%	AIY cell body misplacement	***sax-7***; *nonsense*
						*LGIV: 8,078,943 C→T*, *Q855X*
***hst-6***	*dz134*	X	HS-6-*O*-sulfotransferase	0%		***hst-6***, nonsense
						*LGX: 5,276,053 G→A*, *W127X*
	*dz151*			0%		***hst-6***, missense
						*LGX: 5,276,878 G→A*, *E185K*
	*dz168*			0%		***hst-6***, exon/intron (splice donor);
						*LGX: 5,275,652 G→A*
***hst-3.2***	*dz140*	X	HS-3-*O*-sulfotransferase	60%		***hst-3.2***, exon/intron (splice donor);
						*LGX: 2,923,754 C→T*
	*dz164**^b^*			3%		***hst-3.2***, nonsense
						*LGX: 2,919,308 C→T*, *R138X*
	*dz169**^b^*			0%		***hst-3.2***, missense
						*LGX: 2,923,819 A→T*, *N51K*
	*dz171**^b^*			0%		***hst-3.2***, nonsense
						*LGX: 2,923,827 G→A*, *R49X*
***sqv-6***	*dz165*	V	Xylosyltransferase	25%	sick, Egl (egg laying defective)	***sqv-6*;** exon/intron (splice acceptor), *LGV: 955,016 C→T*
**TBD**	*dz148*		TBD	34%		Complements *hse-5 and pst-1*
**TBD**	*dz147*	V	TBD	**Enh**: 63%	Sma (small)	Fails to complement *ot21*

TBD, to be determined.

a N = 100 in all cases.

b Data from Tecle *et al.* (2013).

The majority of remaining alleles affected genes that are involved in the modification of heparan sulfate (Figure 2, Table 2). Specifically, we isolated three new alleles of the HS-6-*O*-sulfotransferase: a nonsense mutation (*dz134*) is predicted to result in a stop codon after 127 amino acids; a missense mutation (*dz151*) changes a well-conserved negative charge in the 3′ phosphoadenosyl-phophosulfate (PAPS) substrate-binding site (E185K) to a positive charge (Figure 2, Table 2, Figure S3). PAPS represents the universal sulfate donor and is, as co-substrate, required for all sulfation reactions *in vivo*. Finally, *hst-6(dz168)*, a splice donor mutation, is predicted to result in retention of intron 1 and a premature stop after 23 nonhomologous amino acids. In addition, we isolated three point mutant alleles of the HS-3-*O*-sulfotransferase type II, which have been described previously (Tecle *et al.* 2013) (Figure 2, Table 2). A fourth allele *hst-3.2(dz140)* contained a splice donor mutation after exon 1, predicted to result in retention of intron 1 and premature termination. This allele is likely a hypomorphic allele because it only weakly suppressed *kal-1*-dependent branching (Figure 2, Table 2). Furthermore, we isolated one allele of the xylosyltransferase *sqv-6* that harbored a splice acceptor mutation in exon 5 predicted to result in skipping of exon 5, which would lead to a 160-amino-acid in-frame deletion of the xylosyltransferase domain (Figure 1, Table 2). The xylosyltransferase encoded by *sqv-6* (Hwang *et al.* 2003) initiates the polymerization of glycosaminoglycan chains such a HS or chondroitin onto the protein backbone (Figure 1B) (Lindahl and Li 2009). Intriguingly, we found that the allele *dz165* failed to complement a previously identified allele of the PAPS transporter *pst-1(ot20)* (Table S1) (Bhattacharya *et al.* 2009). This nonallelic noncomplementation supports the notion that the two genes act genetically in the same pathway for *kal-1*-dependent branching of AIY neurons and suggests that HS rather than chondroitin is essential for *kal-1* function (Bülow *et al.* 2002; Hudson *et al.* 2006).

### Heparan sulfate proteoglycans act redundantly to pattern the nervous system

**Figure 3:**
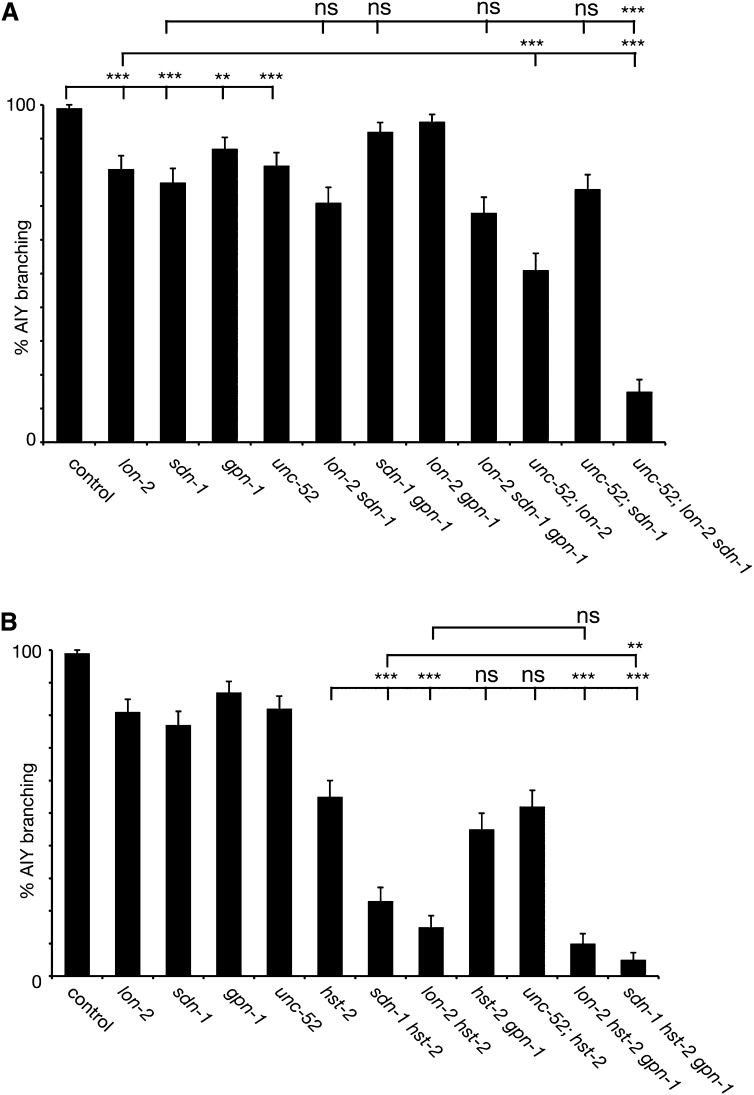
HSPG act redundantly to mediate the *kal-1*-dependent branching in AIY. (A) Genetic analysis of *kal-1*-dependent branching in AIY in heparan sulfate proteoglycan core protein mutants as indicated. Asterisks denote statistical significance: *P < 0.05; **P < 0.005; ***P < 0.0005; ns, not significant in both panels (A) and (B). (B) Genetic analysis of *kal-1*-dependent branching in AIY between heparan sulfate proteoglycan core proteins and the HS-2-*O*-sulfotransferase *hst-2* as indicated. Data for HSPG single mutants are identical to (A) and shown for comparison only.

The repeated identification of mutants in heparan sulfate modification enzymes in the screens as suppressors of the *kal-1*-dependent branching phenotype prompted the question, which HSPG core protein(s) carry the responsible HS chains? The *C. elegans* genome encodes the canonical HSPGs *sdn-1*/syndecan, *gpn-1*/glypican, *lon-2*/glypican, and *unc-52*/perlecan (Bülow and Hobert 2006). We thus tested null mutants in *sdn-1/*syndecan, *lon-2/*glypican, *gpn-1/*glypican, and in a splice variant–specific null allele of *unc-52/*perlecan (Rogalski *et al.* 1993) (Figure 3A). All four HSPG core protein mutants displayed significant suppression of *kal-1*-dependent branching, but surprisingly nowhere similar to mutations in HS modifying enzymes. This suggested that either more than one HSPG or an unidentified HSPG is required for *kal-1*-dependent branching. Alternatively, the function of other splice variants of *unc-52*/perlecan could be sufficient to mediate most of the function required for *kal-1*-dependent branching. To discriminate between these possibilities, we constructed double mutants and found that only the *unc-52(e998)*; *lon-2(e678)* double mutant displayed an enhanced level of suppression (51%; N = 100) that was different from the single mutants or the other double mutants (Figure 3A). This indicated that *lon-2*/glypican and *unc-52*/perlecan act in parallel genetic pathways to mediate *kal-1*-dependent branching in AIY. The *unc-52(e998)*; *lon-2(e678) sdn-1(zh20)* triple mutant further suppressed branching to 15% (n = 100), indicating the presence of an additional parallel genetic pathway. This was surprising because the *lon-2*; *sdn-1* double mutant did not display significantly enhanced suppression compared with either of the single mutants. However, in the absence of *unc-52*/perlecan, lack of both *sdn-1*/syndecan and *lon-2*/glypican at the same time appeared to result in synergistic rather than additive effects. Thus, it appears as if *unc-52*/perlecan can substitute for both *sdn-1*/syndecan and *lon-2*/glypican. Interestingly, we observed similar genetic synergy between HSPG core proteins in a loss of function setting. Specifically, we found that midline guidance of PVQ neurites requires *sdn-1*/syndecan and *lon-2*/glypican redundantly (Figure S4). Taken together, these findings suggest that HSPGs, at least *sdn-1*/syndecan, *lon-2*/glypican and *unc-52*/perlecan, act redundantly to mediate *kal-1*-dependent branching and likely neural development in other cellular contexts.

### Different HS modification patterns may be carried by distinct HSPGs

Mutants in *hst-2* have previously been shown to only moderately suppress the *kal-1*-dependent branching phenotype in contrast to mutants in other modification enzymes such as the HS *C*-5 glucuronyl epimerase *hse-5* and the HS sulfotransferases *hst-6* or *hst-3.2*, which almost completely suppressed the phenotype (Bülow and Hobert 2004; Tecle *et al.* 2013). To better understand the relationship between HSPG core proteins and specific HS modifications, we constructed double and triple mutants between HSPG core proteins and *hst-2*. Interestingly, suppression of the branching observed in the double mutants *sdn-1(zh20) hst-2(ok595)* and *lon-2(e678) hst-2(ok595)* was enhanced when compared with that of the single mutants, whereas that of *unc-52(e998)*; *hst-2(ok595)* was not (Figure 3B). Moreover, we found that genetic removal of *gpn-1*/glypican from the *lon-2(e678) hst-2(ok595)* and *sdn-1(zh20) hst-2(ok595)* double mutants, respectively, further enhanced suppression of *kal-1*-dependent branching (Figure 3B). Collectively, these findings showed that *hst-2* and *sdn-1*/syndecan or *lon-2*/glypican, respectively, act genetically in parallel pathways, and that *hst-2* and *unc-52*/perlecan could be acting in the same genetic pathway to mediate *kal-1*-dependent branching. Finally, these findings indicated a cryptic function for *gpn-1*/glypican in the absence of *lon-2*/glypican and the HS-2-*O*-sulfotransferase *hst-2*. By inference, these findings suggest that different types of HS carried by at least two HSPGs are necessary for the *kal-1*-dependent branching in AIY, one of them being the UNC-52/perlecan and the other being either SDN-1/syndecan or LON-2/glypican. Moreover, 2-*O* sulfated HS may be carried by UNC-52/perlecan.

### HS modifications are required in different tissues to mediate *kal-1*-dependent branching

**Figure 4:**
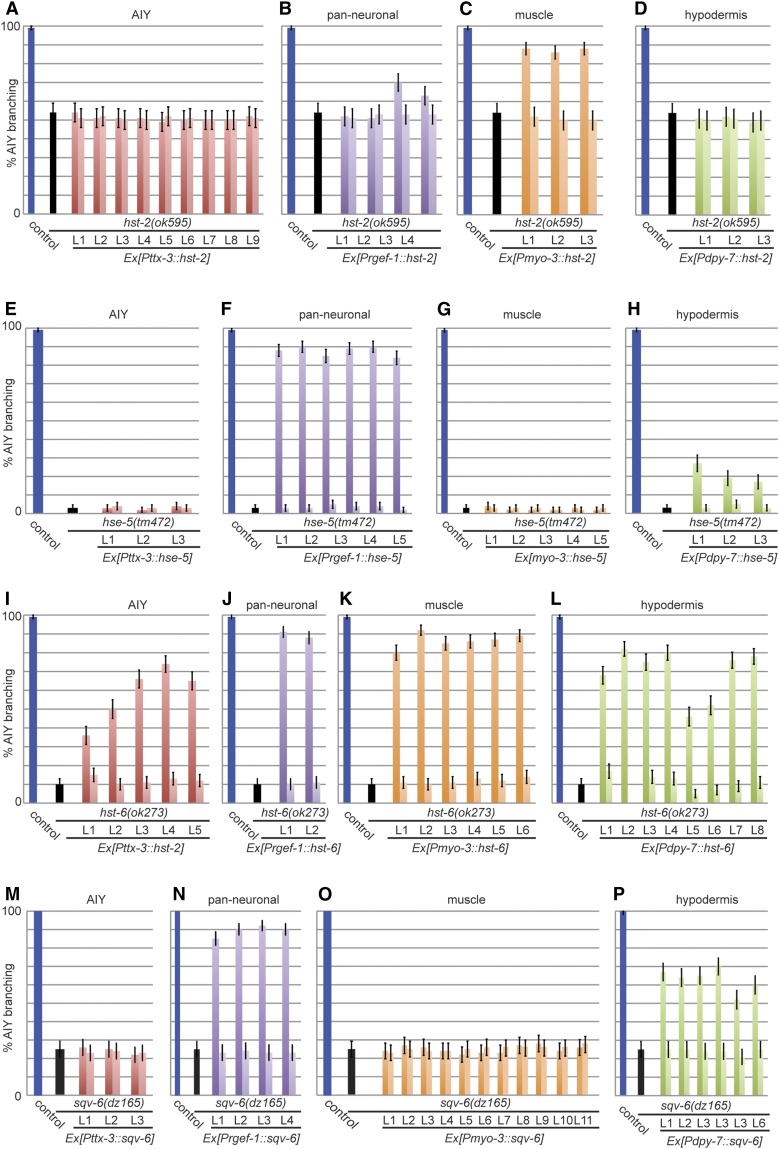
Cell-specific rescue of the *kal-1*-dependent axon branching in heparan sulfate–modifying mutants. (A–D) Rescue of *hst-2(ok595)* with *hst-2* cDNA under heterologous promoters as indicated. In all panels, blue bars indicate the wild-type control (*otIs76 mgIs18(Is[Pttx-3*::*kal-1*, *Pttx-3*::*gfp]*) that displays the completely penetrant *kal-1*-dependent branching phenotype in AIY neurons (Bülow *et al.* 2002); black bars indicate the branching phenotype in the respective mutant and color-coded bars indicate branching in transgenic animals (darker shade) and their nontransgenic siblings (lighter shade). The number of extrachomosomal (*Ex*) transgenic lines (L) is indicated. N = 100 in all cases. *Full rescue was defined as being significantly different from the mutant control and exhibiting 80% or more of branching. Partial rescue was defined as being significantly different from the mutant control and exhibiting less than 80% of branching (n = 100 per transgenic line). (E–H) Rescue of *hst-5(tm472)* with *hse-5* cDNA under heterologous promoters as indicated. (I–L) Rescue of *hst-6(ok273)* with *hst-6* cDNA under heterologous promoters as indicated. (M–P) Rescue of *sqv-6(dz165)* with *sqv-6* cDNA under heterologous promoters as indicated.

The identification of several mutations in HS-modifying enzymes in our genetic screen underscored the importance of HS modifications for *kal-1*-dependent branching (Figure 2). To deconvolute the function of individual modifications, we sought to determine in which tissues HS-modifying enzymes can act to mediate *kal-1*-dependent branching. To this end, we utilized transgenic rescue assays and drove expression of the respective cDNAs under control of heterologous promoters in the hypodermis, in muscle, in neurons, or, specifically, in AIY interneurons. We then determined in which tissue the mutant phenotype, *i.e.*, suppression of the *kal-1*-dependent branching, could be rescued. We discovered that all of the heparan sulfate modifying enzymes (*hst-6*, *hse-5*, and *hst-2*) could nonautonomously rescue the *kal-1*-dependent branching in one or more tissues (Figure 4). Interestingly, expression of *hst-2* in muscle alone was able to rescue the suppression of *kal-1*-dependent branching (with the possible exception of minor rescue when expressed in the nervous system) (Figure 4, A–D). Yet, expression of *hse-5* was only able to rescue significantly when expressed in the nervous system (minor rescue could be attained when *hse-5* was expressed in hypodermal tissues); however, expression of *hse-5* in AIY interneurons was not sufficient (Figure 4, E–H). In contrast, expression of *hst-6* rescued when expressed in any tissue we tested, including in AIY interneurons, although rescue in AIY interneurons was not as robust as when expressed more widely (Figure 4, I–L). Interestingly, similar findings of cell specificity were made for the HS-3*-O*-sulfotransferase *hst-3.2*, which rescued *kal-1*-dependent branching only when expressed in the nervous system or in muscle (Tecle *et al.* 2013).

To determine in which tissue HS is sufficient for *kal-1* function, we performed similar experiments with the *loss of function* (*lof*) mutation in the *sqv-6/*xylosyltransferase, which is required for all HS biosynthesis (Figure 4, M–P). We found that heterologous expression of *sqv-6/*xylosyltransferase in the nervous system or in hypodermal tissues (although not quite as efficiently) can rescue the *kal-1*-dependent branching, whereas expression in AIY or muscle failed to rescue *kal-1*-dependent branching (Figure 4O). We conclude that *sqv-6/*xylosyltransferase in muscle or AIY alone (and by inference HS biosynthesis) is not sufficient for *kal-1*-dependent branching in AIY and that HS from additional cellular sources is required for efficient branch formation. In contrast, expression of *sqv-6/*xylosyltransferase (and by inference HS biosynthesis) in hypodermal and neuronal tissues is under certain experimental circumstances sufficient for *kal-1*-dependent branching of AIY neurons.

Based on these rescue experiments, we propose the existence of at least three HS epitopes with different importance for the *kal-1*-dependent branching in AIY. One epitope is produced by neurons and is modified by *C*-5 epimerization and HS-6-*O* and HS-3-*O*-sulfation. This is supported by rescue of pan-neuronally expressed *hse-5*, *hst-6*, and *hst-3.2*, but not *hst-2*. A second epitope may be produced by the muscle and is modified by HS-2*-O*, HS-3-*O*, and HS-6-*O*-sulfation. This is supported by rescue through muscle-driven expression of *hst-2*, *hst-3.2*, and *hst-6*, but not *hse-5*. A third, possibly less sulfated epitope is produced by the hypodermis and only depends on 6-*O* sulfation. This is supported by hypodermal rescue of *hst-6*, but not *hse-5*, *hse-3.2*, or *hst-2*. It is important to note that these assays do not preclude that any of the epitopes also contain other modifications, but merely indicate that the identified modifications are nondispensable under the experimental conditions.

### HS modifications do not appear to be required for KAL-1 localization

**Figure 5:**
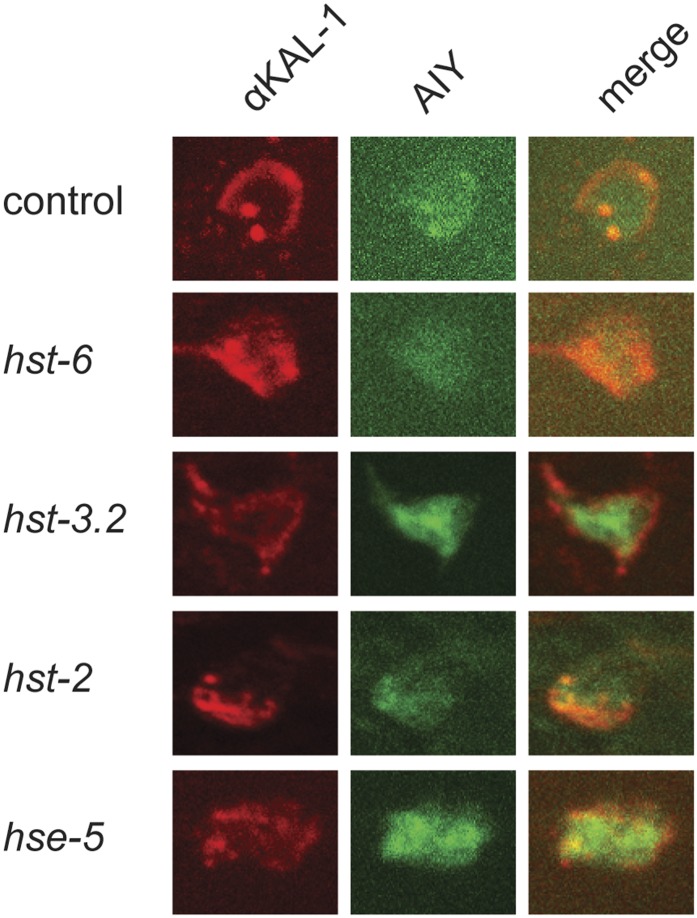
Localization of the KAL-1 is independent of HS modifications. Shown are antibody stains with an ɑKAL-1 antibody (Bülow *et al.* 2002) of KAL-1 expressing animals (*otIs76 mgIs18(Is[Pttx-3*::*kal-1*, *Pttx-3*::*gfp]*) in different genetic backgrounds as indicated. As described (Bülow *et al.* 2002), KAL-1 staining (red) appears to label the cell surface of the AIY interneurons (green) and is not visibly affected in different mutant backgrounds under the experimental conditions.

A possible role of the polyanionic HS could be to bind and localize KAL-1 to the cell surface. Alternatively, specific HS modification patterns could modulate KAL-1 function by determining possible interactions with other factors. To distinguish between these possibilities, we analyzed the localization of KAL-1 expression in different backgrounds mutant for HS-modifying enzymes. In animals in which KAL-1 is misexpressed in AIY neurons, KAL-1 appears localized to the cellular periphery (Figure 5) (Bülow *et al.* 2002). We found the localization of KAL-1 to not be visibly different in any of the mutants, including *hse-5*, *hst-2*, *hst-6*, or *hst-3.2* (Figure 5). Because most mutants individually completely suppress the *kal-1*-dependent branching phenotype in AIY neurons, these data suggest that the major function of HS is not to retain and localize KAL-1 to the cell surface, but rather suggest that HS may be required to mediate how KAL-1 interacts with other factors.

## Discussion

In this study we expanded a screen to identify loci that genetically interact with the Kallmann syndrome disease-causing gene *kal-1*. We identified additional alleles of previously identified mutants, namely *hst-6* (Bülow *et al.* 2002), but also mutants in new genes of the heparan sulfate synthesis and modification machinery, namely *sqv-6*/xylosyltransferase and the HS 3-*O*-sulfotransferase *hst-3.2*. The extended screen also identified genes that are involved in the maintenance of the nervous system, namely *dig-1* and *sax-7*/L1CAM. Our genetic analyses show that several HSPG core proteins act redundantly to mediate *kal-1* function and that those core proteins likely bear distinct HS modification patterns.

### HSPGs act redundantly to mediate the development of the nervous system

Single mutants of the HSPGs only weakly suppressed the *kal-1*-dependent branching. For example, eliminating *lon-2*/glypican (known to function in the hypodermis to regulate migration of HSN motor neurons) (Pedersen *et al.* 2013) or *unc-52*/perlecan (known to be expressed in muscle) (Rogalski *et al.* 1993) or *sdn-1*/syndecan (known to function in the nervous system (Rhiner *et al.* 2005), respectively, did not cause major defects in the formation of *kal-1*-dependent branches. However, double mutants between *unc-52*/perlecan and *lon-2*/glypican did substantially suppress *kal-1*-dependent branching, and suppression was essentially complete upon additional removal of *sdn-1*/syndecan. Similar redundancy was observed in axonal pathfinding of the glutamatergic interneuron PVQ, where we observed that *lon-2*/glypican and *sdn-1*/syndecan act synergistically. The simplest explanation for these results is that HSPGs act redundantly and, if one is not present, the others can partially compensate for its function. This is also consistent with previous observations demonstrating that some of the HS-modifying enzymes, namely *hst-2* and *hst-6*, act in parallel genetic pathways (Bülow and Hobert 2004). Because redundancy between HSPGs has also been observed in ventral closure during gastrulation (Hudson *et al.* 2006), the redundancy of HSPG core proteins may be a more general theme during animal development.

### *kal-1*-dependent branching may require three distinct HS epitopes from different cellular sources

**Figure 6:**
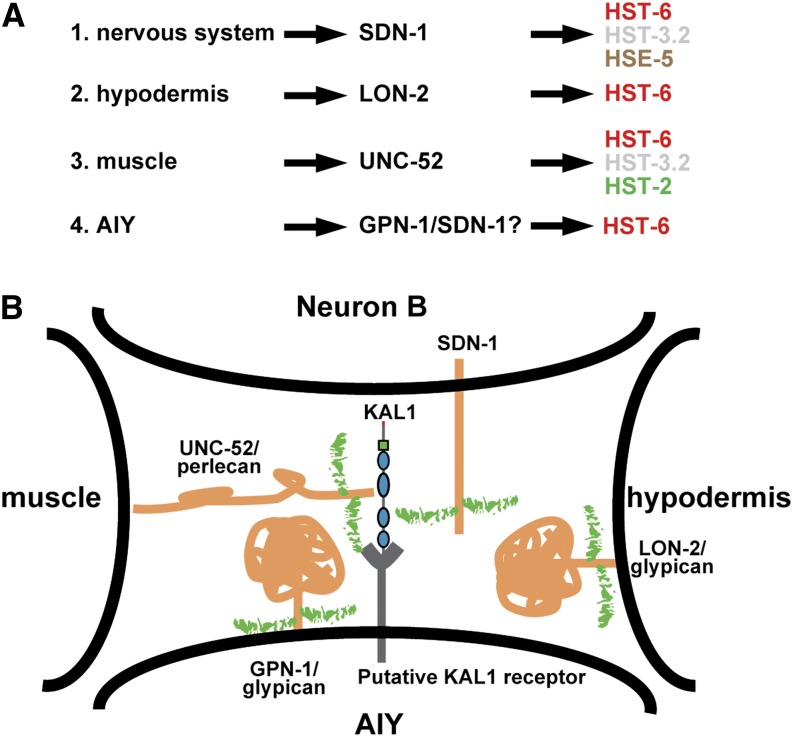
Working model of heparan sulfate–dependent KAL-1-branching. (A) Several pathways are proposed that act genetically in parallel to mediate *kal-1*-dependent branching. Each may require different combinations of heparan sulfate modifications and originate from different tissues. (B) Model of how different heparan sulfate proteoglycans (yellow, with green glycan chains attached) act from several tissues with different HS modification patterns in a highly redundant fashion to allow *kal-1*-dependent branching. Note that SDN-1/syndecan may also be present in AIY neurons, as may be other HSPGs.

An unexpected finding in our studies was that HSPG core proteins act in several parallel genetic pathways and redundantly to mediate *kal-1*-dependent branching. Because *kal-1* requires a distinct set of HS modifications for branching in AIY (6-*O*-, 2-*O*-, and 3-*O*-sulfation and *C-5*-epimerization) (Bülow and Hobert 2004; Tecle *et al.* 2013), one possibility would be that HSPGs from all surrounding tissues carry the same HS modification patterns (required for *kal-1*-dependent branching) and, possibly, that a critical amount is required that one tissue alone would not be able to supply. However, we consider this scenario less likely for the following reasons. First, expression analyses of genes encoding HS modifying enzymes indicate that these genes are differentially expressed in different tissues (Bülow and Hobert 2004), rendering it highly unlikely that HSPGs of different cellular origin bear the same HS modification patterns. Second, direct visualization of defined HS modification patterns in live animals display strikingly specific cellular expression patterns in *C. elegans* (Attreed *et al.* 2012). Thus, based on the data we present here and known expression data for the involved genes, we propose the following model (Figure 6A). UNC-52/perlecan is secreted by the muscle and localized to the extracellular matrix between the hypodermis and the muscle (Moerman *et al.* 1996). Therefore, the sulfated HS epitope that contains 2-*O*-, 3-*O*-, and 6-*O*-sulfation and that is produced by the muscle could be carried by UNC-52/perlecan. Second, *sdn-1*/syndecan has been shown to rescue its mutant phenotypes when expressed pan-neuronally (Rhiner *et al.* 2005), and much of the HS in nematodes is associated with SDN-1/syndecan in the nervous system (Minniti *et al.* 2004). Thus, the highly sulfated HS epitope that contains *C*-5 epimerized and 6-*O*- and 3-*O* sulfated HS and is produced by neurons is most likely carried by SDN-1/syndecan. Third, *lon-2*/glypican has been shown to act in the hypodermis to mediate migration of HSN motor neurons in a HS-dependent manner (Pedersen *et al.* 2013). Therefore, the less sulfated HS epitope that is dependent on HS 6-*O*-sulfation and partially on HS- 2-*O*-sulfation and is produced by the hypodermis may be carried by LON-2/glypican.

A surprising finding was the apparent cell specificity of transgenic rescue for several HS-modifying enzymes such as *hse-5*, *hst-2*, or *hst-3.2* (Tecle *et al.* 2013). In this context, it is important to remember that the transgenic rescue experiments test sufficiency, not necessity, of these genes in the respective tissues. Overexpression of HS-modifying enzymes has been shown to change HS composition (Bülow *et al.* 2008; Kamimura *et al.* 2011). Thus, we cannot exclude the possibility that expressing the enzymes in certain tissues results in the creation of functional HS epitopes that would normally not be present in this tissue. Similarly, we cannot exclude the possibility that the nonautonomous rescue of *hst-6*, but not *hst-2*, mutants is the result of secretion of the enzyme, as has been shown for the vertebrate homologs of *hst-6* in cell culture (Habuchi *et al.* 1998). Nonetheless, the most parsimonious explanation for the genetic data is that distinct epitopes from several tissues are required and that the interactions between all the cells and tissues are necessary for efficient induction of *kal-1*-dependent branches

### A three-dimensional HS scaffold mediates intercellular communication

Our detailed analysis of the genetic interaction between *kal-1* and HS suggest a model in which HS forms a three-dimensional scaffold originating from different tissues to function with KAL-1/anosmin-1 (Figure 6B). How could HSPGs from muscle, hypodermis, or neurons mediate branching in AIY? One possibility could be that secreted UNC-52/perlecan is deposited in the basement membrane during early embryonic development when the distances between the cells are small. Moreover, syndecans and glypicans could be shed from the cell surface (Bernfield *et al.* 1999) and could thus also act at a distance. Alternatively, but not mutually exclusive, HSPGs have been shown to act *in trans* to other cells to regulate TGFβ-signaling or VEGF signaling (Kramer and Yost 2002; Jakobsson *et al.* 2006).

How does HS mediate KAL-1 function? One possibility is that HS could be acting as co-receptor for KAL-1 to enhance its signaling through other receptor(s) by forming a multiprotein complex between one or more co-receptors and ligand(s). For example, KAL1/anosmin-1 binds FGFR1 in an HS-dependent manner *in vitro* (Hu *et al.* 2009) and genetic studies in worms established that EGL-15/FGFR is required in a context-dependent manner for *kal-1*/anosmin-1 function *in vivo* (Tornberg *et al.* 2011). Alternatively, HS may modulate the binding of additional factors to the complex depending on the presence of HS, or may control the distribution of KAL-1 in the ECM to limit diffusion away from AIY. Our findings support the former, because distribution of KAL-1/anosmin-1 is not visibly affected based on antibody stains in different mutant backgrounds (Figure 5). The HS scaffold formed by HSPGs with distinct epitopes from different tissues may coordinate the interactions of KAL-1 with several factors. This scaffold is acting in a highly redundant fashion, possibly on two levels. Even in the absence of one or two HSPGs, the remaining HSPGs may be able to provide sufficient function to maintain a scaffold, even if it is not entirely normal. Another level of redundancy may exist with regard to HS epitope(s) that could originate from diverse tissues as long as these contain the appropriate combination and arrangement of modification patterns for function. In either case, HS epitopes from several tissues and the interactions between all the cells and tissues are necessary to create a functional three-dimensional HS scaffold that mediates *kal-1*-dependent branching.

### Heparan sulfates and Kallmann syndrome

Guided by our work with *C. elegans*, we have previously identified mutations in the HS 6-*O*-sulfotransferase HS6ST1 in patients with Kallmann syndrome/idiopathic hypogonadotrophic hypogonadism (Tornberg *et al.* 2011). *In vitro* studies showed that the identified mutations affected the enzymatic activity of HS6ST1 *in vitro* and *in vivo*. In this cohort of patients, mutations were also identified in the FGFR1, lending support to an oligogenic mode of inheritance for KS/nIHH. This was not unprecedented, because mutations in more than one gene were also identified in other KS/nIHH patients, also supporting the hypothesis of an oligogenic mode of inheritance (Sykiotis *et al.* 2010). Clearly, the FGFR signaling pathway plays a central role in the pathogenesis of KS/nIHH as mutations in several interacting genes have been identified (including HS6OST1, SPRY4, IL17RD, DUSP6, FGF17, and FLRT3) (Tornberg *et al.* 2011; Miraoui *et al.* 2013). Because different HS modification patterns are crucial for FGFR signaling and function (Guimond and Turnbull 1999), all the genes involved in the biosynthesis and modification of heparan sulfates as well as the other genes are candidates to be mutated in still elusive cases of Kallmann syndrome and IHH. Mutations in these novel genes may not be causing the syndrome individually, but rather could contribute to KS/nIHH in conjunction with one or more additional genes.

## Supplementary Material

Supporting Information
