# Supplementary material for: Complex Cooperative Functions of Heparan Sulfate Proteoglycans Shape Nervous System Development in Caenorhabditis elegans
Source: G3 (Bethesda). 2014 Aug 5;4(10):1859–70. doi: 10.1534/g3.114.012591 (PMC4199693; doi:10.1534/g3.114.012591)
Supplement: Supporting Information [file supp_4_10_1859__index.html]

Complex Cooperative Functions of Heparan Sulfate Proteoglycans Shape Nervous System Development in Caenorhabditis elegans — Supporting Information 

# Complex Cooperative Functions of Heparan Sulfate Proteoglycans Shape Nervous System Development in *Caenorhabditis elegans*

## Supporting Information for Díaz-Balzac *et al.*, 2014

**Files in this Data Supplement:**

- Supporting Information - Tables S1-S2, Figures S1-S4, and Supporting Information References (PDF, 743 KB)
- Table S1 - Summary of genetic experiments for X-linkage and complementation. (PDF, 78 KB)
- Table S2 - List of Transgenic strains. (PDF, 30 KB)
- Figure S1 - Phylogenetic Tree of KAL1/anosmin-1 proteins. (PDF, 345 KB)
- Figure S2 - *dig-1* cell positioning defects in AIY. (PDF, 129 KB)
- Figure S3 - Molecular lesions in *hst-6* and *sqv-6*. (PDF, 165 KB)
- Figure S4 - HSPG act redundantly to mediate midline patterning of the PVQ axons. (PDF, 44 KB)
