# Supplementary material for: Complex Cooperative Functions of Heparan Sulfate Proteoglycans Shape Nervous System Development in Caenorhabditis elegans
Source: G3 (Bethesda). 2014 Aug 5;4(10):1859–70. doi: 10.1534/g3.114.012591 (PMC4199693; doi:10.1534/g3.114.012591)
Supplement: Supporting Information [file supp_g3.114.012591_012591SI.pdf]

G3 · Genes | Genomes | Genetics

Complex cooperative functions of heparan sulfate proteoglycans shape nervous system development in *C. elegans*

Carlos A. Díaz-Balzac[1], Maria I. Lázaro-Peña[1], Eillen Tecle[1], Nathali Gomez[1], and Hannes E. Bülow[1,2]*

Department of Genetics[1] and Dominick P. Purpura Department of Neuroscience[2]

Albert Einstein College of Medicine

Bronx, New York, 10461

Correspondence to: hannes.buelow@einstein.yu.edu

* corresponding author:

Telephone 718 430 3621

Fax 718 430 8778

e-mail: hannes.buelow@einstein.yu.edu

DOI: 10.1534/g3.114.012591

Table S1 Summary of genetic experiments for X-linkage and complementation

**Data for mutants with cell body misplacement**		
**Genotype**	**% misplacement**	**N**
dig-1(dz152)/+; *otIs76mgIs18/mgIs18; him-5(e1490)/+; otIs35/0 (♂)*	4	24
dig-1(dz136)/+; *otIs76mgIs18/mgIs18; him-5(e1490)/+; otIs35/0 (♂)*	0	15
dig-1(dz145)/+; *otIs76mgIs18/mgIs18; him-5(e1490)/+; otIs35/0 (♂)*	0	14
dig-1(dz154)/+; *otIs76mgIs18/mgIs18; him-5(e1490)/+; otIs35/0 (♂)*	0	32
dig-1(dz155)/+; *otIs76mgIs18/mgIs18; him-5(e1490)/+; otIs35/0 (♂)*	0	36
dig-1(dz152)/+; *otIs76mgIs18/mgIs18; him-5(e1490)/+; otIs35/+ (⚥)*	0	30
dig-1(dz136)/+; *otIs76mgIs18/mgIs18; him-5(e1490)/+; otIs35/+ (⚥)*	0	15
dig-1(dz145)/+; *otIs76mgIs18/mgIs18; him-5(e1490)/+; otIs35/+ (⚥)*	0	37
dig-1(dz154)/+; *otIs76mgIs18/mgIs18; him-5(e1490)/+; otIs35/+ (⚥)*	0	32
dig-1(dz155)/+; *otIs76mgIs18/mgIs18; him-5(e1490)/+; otIs35/+ (⚥)*	0	32
dig-1(dz152)/dig-1(ky388); *otIs76mgIs18/mgIs18; him-5(e1490)/+; otIs35/+ (⚥)*	76	50
dig-1(dz152)/dig-1(n1321); *otIs76mgIs18/mgIs18; him-5(e1490)/+; otIs35/+ (⚥)*	86	50
dig-1(dz136)/dig-1(dz152); *otIs76mgIs18/mgIs18; him-5(e1490)/+; otIs35/+ (⚥)*	16	75
dig-1(dz145)/dig-1(dz155); *otIs76mgIs18/mgIs18; him-5(e1490)/+; otIs35/+ (⚥)*	70	57
dig-1(dz154)/dig-1(dz152); *otIs76mgIs18/mgIs18; him-5(e1490)/+; otIs35/+ (⚥)*	92	36
dig-1(dz155)/dig-1(dz152); *otIs76mgIs18/mgIs18; him-5(e1490)/+; otIs35/+ (⚥)*	95	37
dig-1(dz152); *otIs76mgIs18; rhEx40 (Rescuing array) (⚥)*	5	100
**Data for mutants that suppress *kal-1*-dependent branching**	**% branching**	**N**
dig-1(dz136)/dig-1(dz152); *otIs76mgIs18/mgIs18; him-5(e1490)/+; otIs35/+ (⚥)*	69	75
otIs76mgIs18/mgIs18; him-5(e1490)/+; otIs35 ***hst-6(dz134)***/0 *(♂)*	19	16
otIs76mgIs18/mgIs18; him-5(e1490)/+; otIs35 ***hst-6(dz151)***/0 *(♂)*	10	30
otIs76mgIs18/mgIs18; him-5(e1490)/+; otIs35 ***hst-6(dz168)***/0 *(♂)*	9	55
otIs76mgIs18/mgIs18; him-5(e1490)/+; otIs35 ***hst-6(dz134)***/+ *(⚥)*	100	13
otIs76mgIs18/mgIs18; him-5(e1490)/+; otIs35 ***hst-6(dz151)***/+ *(⚥)*	96	24
otIs76mgIs18/mgIs18; him-5(e1490)/+; otIs35 ***hst-6(dz168)***/+ *(⚥)*	92	51
otIs76mgIs18/mgIs18; him-5(e1490)/+; ***+ hst-6(ok273)/otIs35 hst-6(dz134)*** *(⚥)*	4	50
otIs76mgIs18/mgIs18; him-5(e1490)/+; ***+ hst-6(ok273)/otIs35 hst-6(dz151)*** *(⚥)*	5	22
otIs76mgIs18/mgIs18; him-5(e1490)/+; ***+ hst-6(ok273)/otIs35 hst-6(dz168)*** *(⚥)*	6	50
otIs76mgIs18/mgIs18; him-5(e1490)/+; otIs35 ***hst-3.2(dz140)***/0 *(♂)*	95	44
otIs76mgIs18/mgIs18; him-5(e1490)/+; otIs35 ***hst-3.2(dz140)***/+ *(⚥)*	100	45
otIs76mgIs18/mgIs18; him-5(e1490)/+; ***+ hst-3.2(dz171)/otIs35 hst-3.2(dz140)*** *(⚥)*	12	50
otIs76mgIs18/mgIs18; ***sqv-6(dz165)***/+; *otIs35/0 (♂)*	97	30
otIs76mgIs18/mgIs18; ***sqv-6(dz165)***/+; *otIs35/+ (⚥)*	90	40
otIs76mgIs18/mgIs18; ***sqv-6(dz165)***/*pst-1(ot20); otIs35/+ (⚥)*	20	20
otIs76mgIs18/mgIs18; ***sqv-6(dz165)***/*hse-5(tm472); otIs35/+ (⚥)*	100	20
dz148/+; otIs76mgIs18/mgIs18; otIs35/0 (♂)	88	26
dz148/+; otIs76mgIs18/mgIs18; otIs35/+ (⚥)	91	34
**Data for mutants that enhance *kal-1*-dependent branching**	**% enhancement**	**N**
otIs76mgIs18/mgIs18; ***dz147***/+; *otIs35/0 (♂)*	0	23
otIs76mgIs18/mgIs18; ***dz147***/+; *otIs35/+ (⚥)*	0	24
otIs77/+; mgIs18/mgIs18; ***dz147/ot21***; *otIs35/+ (⚥)*	56	50

Table S2   List of Transgenic strains

Strain name	Constructs	Genotype
EB1543	*pttx-3::hst-6 and pmyo-3::mCherry*	*dzEx725; otIs76mgIs18IV; hst-6(ok273)X*
EB1544	*pttx-3::hst-6 and pmyo-3::mCherry*	*dzEx726; otIs76mgIs18IV; hst-6(ok273)X*
EB1691	*pttx-3::hst-6 and pmyo-3::mCherry*	*dzEx818; otIs76mgIs18IV; hst-6(ok273)X*
EB1692	*pttx-3::hst-6 and pmyo-3::mCherry*	*dzEx819; otIs76mgIs18IV; hst-6(ok273)X*
EB1693	*pttx-3::hst-6 and pmyo-3::mCherry*	*dzEx820; otIs76mgIs18IV; hst-6(ok273)X*
EB1698	*prgef-1::hst-6 and pmyo-3::mCherry*	*dzEx825; otIs76mgIs18IV; hst-6(ok273)X*
EB1699	*prgef-1::hst-6 and pmyo-3::mCherry*	*dzEx826; otIs76mgIs18IV; hst-6(ok273)X*
EB1489	*pdpy-7::hst-6 and pmyo-3::mCherry*	*dzEx685; otIs76mgIs18IV; hst-6(ok273)X*
EB1490	*pdpy-7::hst-6 and pmyo-3::mCherry*	*dzEx686; otIs76mgIs18IV; hst-6(ok273)X*
EB1491	*pdpy-7::hst-6 and pmyo-3::mCherry*	*dzEx687; otIs76mgIs18IV; hst-6(ok273)X*
EB2427	*pdpy-7::hst-6 and pmyo-3::mCherry*	*dzEx826; otIs76mgIs18IV; hst-6(ok273)X*
EB1492	*pdpy-7::hst-6 and pmyo-3::mCherry*	*dzEx688; otIs76mgIs18IV; hst-6(ok273)X*
EB1488	*pdpy-7::hst-6 and pmyo-3::mCherry*	*dzEx684; otIs76mgIs18IV; hst-6(ok273)X*
EB2428	*pdpy-7::hst-6 and pmyo-3::mCherry*	*dzEx826; otIs76mgIs18IV; hst-6(ok273)X*
EB1487	*pdpy-7::hst-6 and pmyo-3::mCherry*	*dzEx683; otIs76mgIs18IV; hst-6(ok273)X*
EB2429	*pmyo-3::hst-6 and pmyo-3::mCherry*	*dzEx683; otIs76mgIs18IV; hst-6(ok273)X*
EB1694	*pmyo-3::hst-6 and pmyo-3::mCherry*	*dzEx821; otIs76mgIs18IV; hst-6(ok273)X*
EB1486	*pmyo-3::hst-6 and pmyo-3::mCherry*	*dzEx682; otIs76mgIs18IV; hst-6(ok273)X*
EB2430	*pmyo-3::hst-6 and pmyo-3::mCherry*	*dzEx683; otIs76mgIs18IV; hst-6(ok273)X*
EB2431	*pmyo-3::hst-6 and pmyo-3::mCherry*	*dzEx683; otIs76mgIs18IV; hst-6(ok273)X*
EB1485	*pmyo-3::hst-6 and pmyo-3::mCherry*	*dzEx681; otIs76mgIs18IV; hst-6(ok273)X*
EB1700	*pdpy-7::hse-5 and pmyo-3::mCherry*	*dzEx827; hse-5(tm472)III; otIs76mgIs18IV*
EB1701	*pdpy-7::hse-5 and pmyo-3::mCherry*	*dzEx828; hse-5(tm472)III; otIs76mgIs18IV*
EB1702	*pdpy-7::hse-5 and pmyo-3::mCherry*	*dzEx829; hse-5(tm472)III; otIs76mgIs18IV*
EB1551	*pmyo-3::hse-5 and pmyo-3::mCherry*	*dzEx731; hse-5(tm472)III; otIs76mgIs18IV*
EB1789	*pmyo-3::hse-5 and pmyo-3::mCherry*	*dzEx883; hse-5(tm472)III; otIs76mgIs18IV*
EB1790	*pmyo-3::hse-5 and pmyo-3::mCherry*	*dzEx884; hse-5(tm472)III; otIs76mgIs18IV*
EB1791	*pmyo-3::hse-5 and pmyo-3::mCherry*	*dzEx885; hse-5(tm472)III; otIs76mgIs18IV*
EB1792	*pmyo-3::hse-5 and pmyo-3::mCherry*	*dzEx886; hse-5(tm472)III; otIs76mgIs18IV*
EB1793	*pmyo-3::hse-5 and pmyo-3::mCherry*	*dzEx887; hse-5(tm472)III; otIs76mgIs18IV*
EB1730	*pttx-3::hst-2 and pmyo-3::mCherry*	*dzEx838; otIs76mgIs18IV; hst-2(ok595)X*
EB1731	*pttx-3::hst-2 and pmyo-3::mCherry*	*dzEx839; otIs76mgIs18IV; hst-2(ok595)X*
EB1732	*pttx-3::hst-2 and pmyo-3::mCherry*	*dzEx840; otIs76mgIs18IV; hst-2(ok595)X*
EB1733	*pttx-3::hst-2 and pmyo-3::mCherry*	*dzEx841; otIs76mgIs18IV; hst-2(ok595)X*
EB1734	*pttx-3::hst-2 and pmyo-3::mCherry*	*dzEx842; otIs76mgIs18IV; hst-2(ok595)X*
EB1735	*pttx-3::hst-2 and pmyo-3::mCherry*	*dzEx843; otIs76mgIs18IV; hst-2(ok595)X*
EB1736	*pttx-3::hst-2 and pmyo-3::mCherry*	*dzEx844; otIs76mgIs18IV; hst-2(ok595)X*
EB1737	*pttx-3::hst-2 and pmyo-3::mCherry*	*dzEx845; otIs76mgIs18IV; hst-2(ok595)X*
EB1738	*pttx-3::hst-2 and pmyo-3::mCherry*	*dzEx846; otIs76mgIs18IV; hst-2(ok595)X*
EB1739	*pttx-3::hst-2 and pmyo-3::mCherry*	*dzEx847; otIs76mgIs18IV; hst-2(ok595)X*
EB1742	*prgef-1::hst-2 and pmyo-3::mCherry*	*dzEx848; otIs76mgIs18IV; hst-2(ok595)X*
EB1743	*prgef-1::hst-2 and pmyo-3::mCherry*	*dzEx849; otIs76mgIs18IV; hst-2(ok595)X*
EB1744	*prgef-1::hst-2 and pmyo-3::mCherry*	*dzEx850; otIs76mgIs18IV; hst-2(ok595)X*
EB1745	*prgef-1::hst-2 and pmyo-3::mCherry*	*dzEx851; otIs76mgIs18IV; hst-2(ok595)X*
EB1749	*pdpy-7::hst-2 and pmyo-3::mCherry*	*dzEx854; otIs76mgIs18IV; hst-2(ok595)X*
EB1818	*pdpy-7::hst-2 and pmyo-3::mCherry*	*dzEx912; otIs76mgIs18IV; hst-2(ok595)X*
EB1819	*pdpy-7::hst-2 and pmyo-3::mCherry*	*dzEx913; otIs76mgIs18IV; hst-2(ok595)X*
EB1747	*pmyo-3::hst-2 and pmyo-3::mCherry*	*dzEx852; otIs76mgIs18IV; hst-2(ok595)X*
EB1748	*pmyo-3::hst-2 and pmyo-3::mCherry*	*dzEx853; otIs76mgIs18IV; hst-2(ok595)X*
EB1823	*pmyo-3::hst-2 and pmyo-3::mCherry*	*dzEx915; otIs76mgIs18IV; hst-2(ok595)X*
EB1756	*pttx-3::hse-5 and pmyo-3::mCherry*	*dzEx858; hse-5(tm472)III; otIs76mgIs18IV*
EB1757	*pttx-3::hse-5 and pmyo-3::mCherry*	*dzEx859; hse-5(tm472)III; otIs76mgIs18IV*
EB1758	*pttx-3::hse-5 and pmyo-3::mCherry*	*dzEx860; hse-5(tm472)III; otIs76mgIs18IV*
EB1750	*prgef-1::hse-5 and pmyo-3::mCherry*	*dzEx855; hse-5(tm472)III; otIs76mgIs18IV*
EB1754	*prgef-1::hse-5 and pmyo-3::mCherry*	*dzEx856; hse-5(tm472)III; otIs76mgIs18IV*

EB1755	*prgef-1::hse-5 and pmyo-3::mCherry*	*dzEx857; hse-5(tm472)III; otIs76mgIs18IV*
EB1786	*prgef-1::hse-5 and pmyo-3::mCherry*	*dzEx880; hse-5(tm472)III; otIs76mgIs18IV*
EB1787	*prgef-1::hse-5 and pmyo-3::mCherry*	*dzEx881; hse-5(tm472)III; otIs76mgIs18IV*
EB1788	*prgef-1::hse-5 and pmyo-3::mCherry*	*dzEx882; hse-5(tm472)III; otIs76mgIs18IV*
EB2396	*prgef-1::sqv-6 and pmyo-3::mCherry*	*dzEx1337; otIs76mgIs18IV; sqv-6(dz165)X*
EB2397	*prgef-1::sqv-6 and pmyo-3::mCherry*	*dzEx1338; otIs76mgIs18IV; sqv-6(dz165)X*
EB2398	*prgef-1::sqv-6 and pmyo-3::mCherry*	*dzEx1339; otIs76mgIs18IV; sqv-6(dz165)X*
EB2399	*prgef-1::sqv-6 and pmyo-3::mCherry*	*dzEx1340; otIs76mgIs18IV; sqv-6(dz165)X*
EB2400	*pdpy-7::sqv-6 and pmyo-3::mCherry*	*dzEx1341; otIs76mgIs18IV; sqv-6(dz165)X*
EB2401	*pdpy-7::sqv-6 and pmyo-3::mCherry*	*dzEx1342; otIs76mgIs18IV; sqv-6(dz165)X*
EB2402	*pdpy-7::sqv-6 and pmyo-3::mCherry*	*dzEx1343; otIs76mgIs18IV; sqv-6(dz165)X*
EB2403	*pdpy-7::sqv-6 and pmyo-3::mCherry*	*dzEx1344; otIs76mgIs18IV; sqv-6(dz165)X*
EB2404	*pdpy-7::sqv-6 and pmyo-3::mCherry*	*dzEx1345; otIs76mgIs18IV; sqv-6(dz165)X*
EB2405	*pdpy-7::sqv-6 and pmyo-3::mCherry*	*dzEx1346; otIs76mgIs18IV; sqv-6(dz165)X*
EB2406	*pmyo-3::sqv-6 and pmyo-3::mCherry*	*dzEx1347; otIs76mgIs18IV; sqv-6(dz165)X*
EB2407	*pmyo-3::sqv-6 and pmyo-3::mCherry*	*dzEx1348; otIs76mgIs18IV; sqv-6(dz165)X*
EB2408	*pmyo-3::sqv-6 and pmyo-3::mCherry*	*dzEx1349; otIs76mgIs18IV; sqv-6(dz165)X*
EB2409	*pmyo-3::sqv-6 and pmyo-3::mCherry*	*dzEx1350; otIs76mgIs18IV; sqv-6(dz165)X*
EB2410	*pmyo-3::sqv-6 and pmyo-3::mCherry*	*dzEx1351; otIs76mgIs18IV; sqv-6(dz165)X*
EB2411	*pmyo-3::sqv-6 and pmyo-3::mCherry*	*dzEx1352; otIs76mgIs18IV; sqv-6(dz165)X*
EB2412	*pmyo-3::sqv-6 and pmyo-3::mCherry*	*dzEx1353; otIs76mgIs18IV; sqv-6(dz165)X*
EB2413	*pmyo-3::sqv-6 and pmyo-3::mCherry*	*dzEx1354; otIs76mgIs18IV; sqv-6(dz165)X*
EB2414	*pmyo-3::sqv-6 and pmyo-3::mCherry*	*dzEx1355; otIs76mgIs18IV; sqv-6(dz165)X*
EB2415	*pmyo-3::sqv-6 and pmyo-3::mCherry*	*dzEx1356; otIs76mgIs18IV; sqv-6(dz165)X*
EB2416	*pmyo-3::sqv-6 and pmyo-3::mCherry*	*dzEx1357; otIs76mgIs18IV; sqv-6(dz165)X*
EB2417	*pttx-3::sqv-6 and pmyo-3::mCherry*	*dzEx1358; otIs76mgIs18IV; sqv-6(dz165)X*
EB2418	*pttx-3::sqv-6 and pmyo-3::mCherry*	*dzEx1359; otIs76mgIs18IV; sqv-6(dz165)X*
EB2419	*pttx-3::sqv-6 and pmyo-3::mCherry*	*dzEx1360; otIs76mgIs18IV; sqv-6(dz165)X*

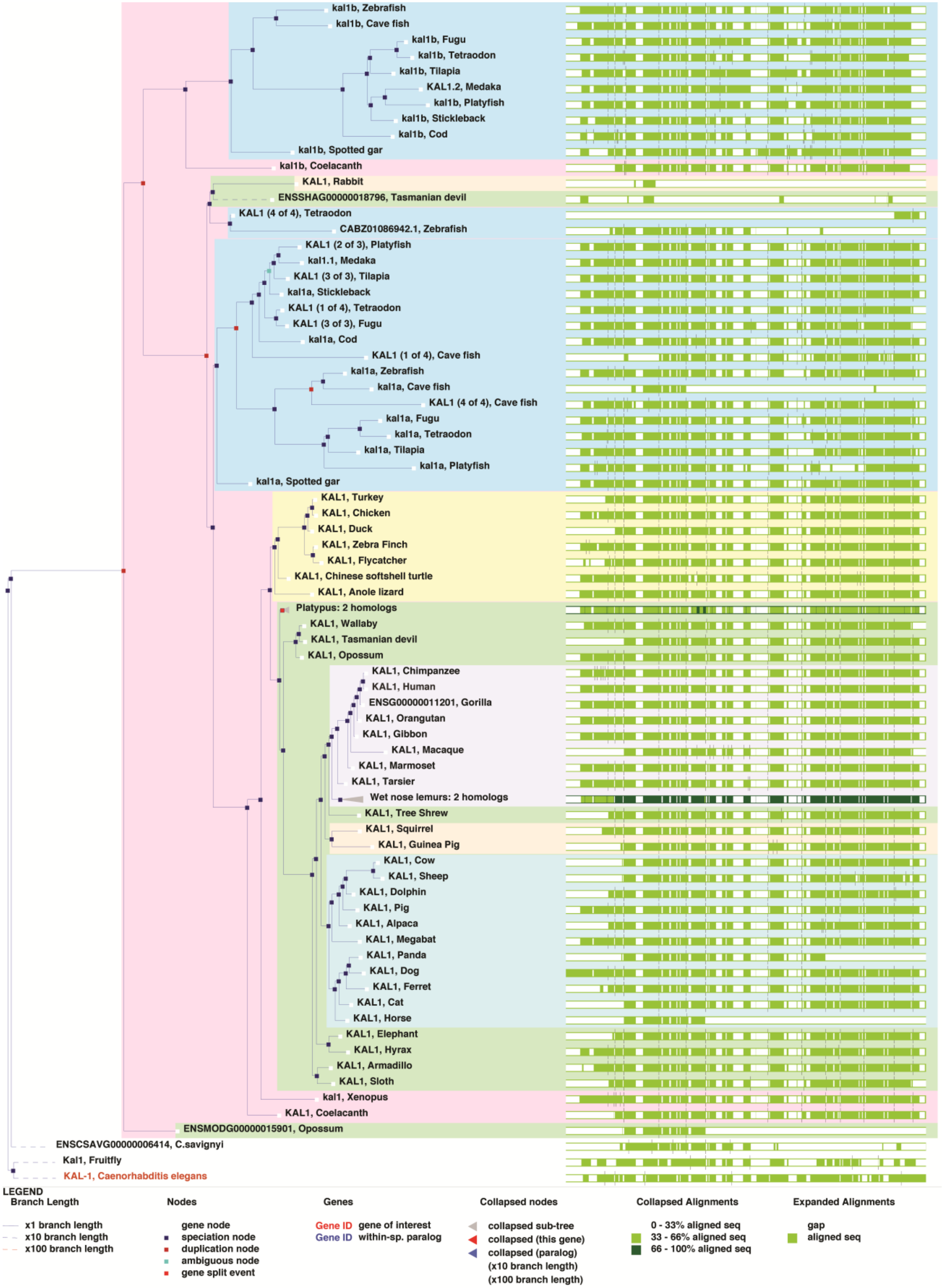
kal1b, Zebrafish
kal1b, Cave fish
kal1b, Fugu
kal1b, Tetraodon
kal1b, Tilapia
KAL1.2, Medaka
kal1b, Platyfish
kal1b, Stickleback
kal1b, Cod
kal1b, Spotted gar
kal1b, Coelacanth
KAL1, Rabbit
ENSSHAG00000018796, Tasmanian devil
KAL1 (4 of 4), Tetraodon
CABZ01086942.1, Zebrafish
KAL1 (2 of 3), Platyfish
kal1.1, Medaka
KAL1 (3 of 3), Tilapia
kal1a, Stickleback
KAL1 (1 of 4), Tetraodon
KAL1 (3 of 3), Fugu
kal1a, Cod
KAL1 (1 of 4), Cave fish
kal1a, Zebrafish
kal1a, Cave fish
KAL1 (4 of 4), Cave fish
kal1a, Fugu
kal1a, Tetraodon
kal1a, Tilapia
kal1a, Platyfish
kal1a, Spotted gar
KAL1, Turkey
KAL1, Chicken
KAL1, Duck
KAL1, Zebra Finch
KAL1, Flycatcher
KAL1, Chinese softshell turtle
KAL1, Anole lizard
Platypus: 2 homologs
KAL1, Wallaby
KAL1, Tasmanian devil
KAL1, Opossum
KAL1, Chimpanzee
KAL1, Human
ENSG00000011201, Gorilla
KAL1, Orangutan
KAL1, Gibbon
KAL1, Macaque
KAL1, Marmoset
KAL1, Tarsier
Wet nose lemurs: 2 homologs
KAL1, Tree Shrew
KAL1, Squirrel
KAL1, Guinea Pig
KAL1, Cow
KAL1, Sheep
KAL1, Dolphin
KAL1, Pig
KAL1, Alpaca
KAL1, Megabat
KAL1, Panda
KAL1, Dog
KAL1, Ferret
KAL1, Cat
KAL1, Horse
KAL1, Elephant
KAL1, Hyrax
KAL1, Armadillo
KAL1, Sloth
kal1, Xenopus
KAL1, Coelacanth
ENSMODG00000015901, Opossum
ENSCSAVG00000006414, C.savignyi
Kal1, Fruitfly
KAL-1, Caenorhabditis elegans
LEGEND
Branch Length
x1 branch length
x10 branch length
x100 branch length
Nodes
gene node
speciation node
duplication node
ambiguous node
gene split event
Genes
Gene ID gene of interest
Gene ID within-sp. paralog
Collapsed nodes
collapsed sub-tree
collapsed (this gene)
collapsed (paralog)
(x10 branch length)
(x100 branch length)
Collapsed Alignments
0 - 33% aligned seq
33 - 66% aligned seq
66 - 100% aligned seq
Expanded Alignments
gap
aligned seq

Figure S1 Phylogenetic Tree of KAL1/anosmin-1 proteins. Phylogenetic tree of KAL1/anosmin-1 obtained from http://www.ensembl.org. Accession number for this tree is ENSGT00440000033720. *C. elegans* KAL-1 is marked in red.

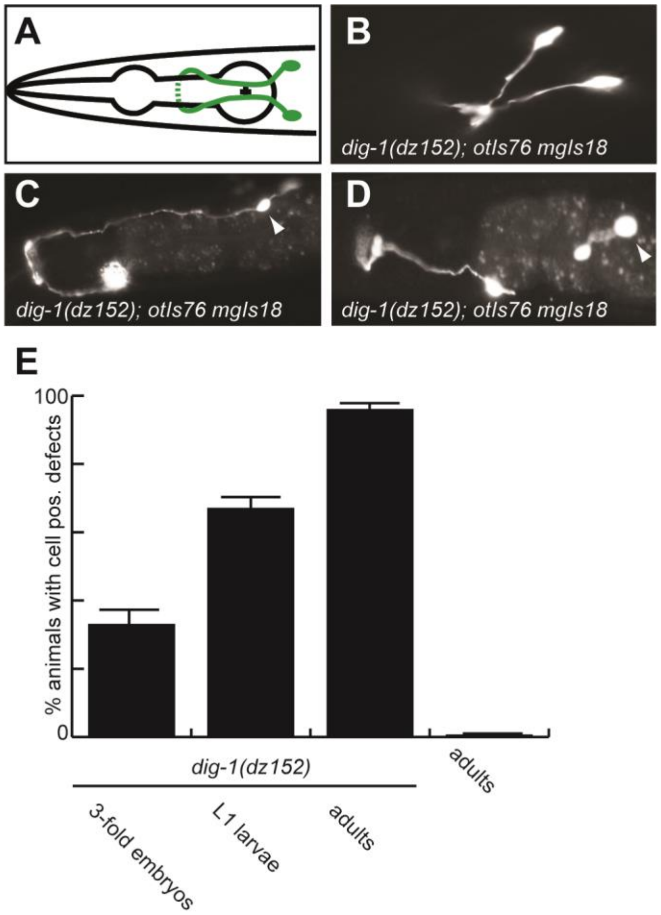

Figure S2 ***dig-1*** **cell positioning defects in AIY.**

A-D: Representative images of *dig-1* defects in AIY. Diagram of a ventral view of AIY in an adult wild type animal (A), *dig-1* mediated suppression of the *kal-1*-dependent branching in AIY (B), *dig-1* suppression of the *kal-1*-dependent branching in AIY and mispositioning of the AIY cell body (C), and mispositioning of the AIY cell body in a *dig-1* mutant independent of the *kal-1 gof* branching in AIY (D). Arrowheads indicate mispositioned AIY cell bodies.

E. Quantification of animals with cell positioning defects at the developmental stages and genotypes indicated All experiments were performed in a AIY *otIs76 mgIs18(Is[Pttx-3::kal-1, Pttx-3::gfp]* background. Error bars indicate the standard error of proportion. N≥100 in all cases.

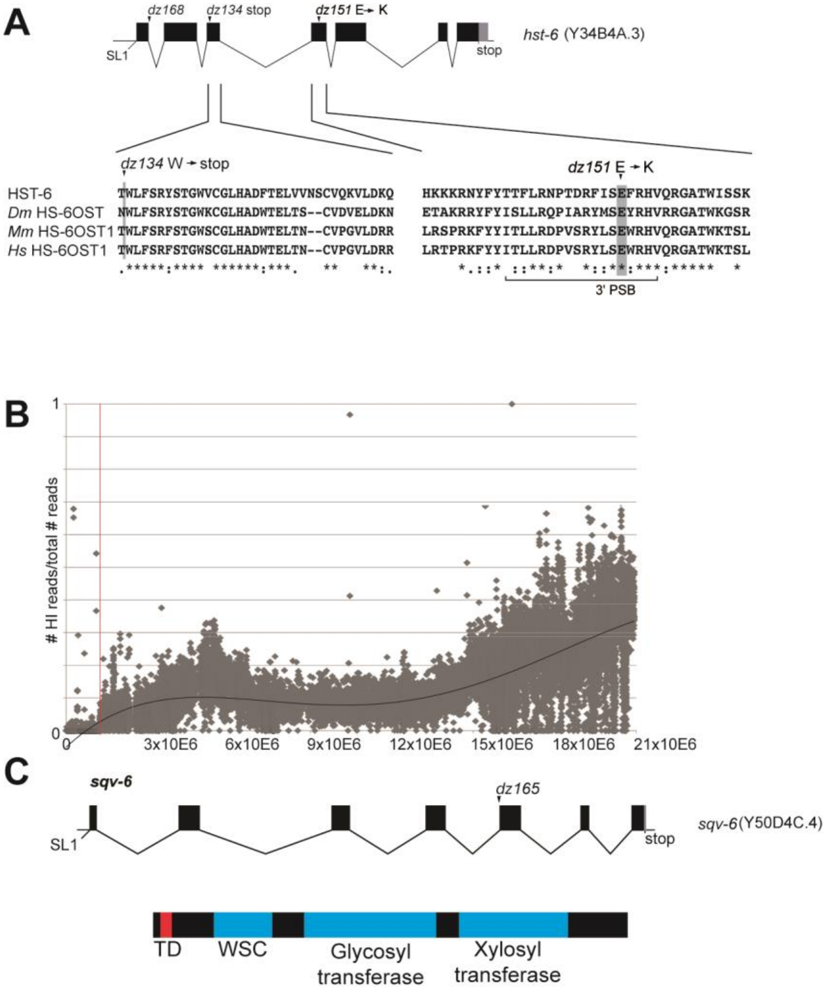

Figure S3 Molecular lesions in *hst-6* and *sqv-6.*

A. *hst-6* gene structure with molecular lesions of *dz134*, and *dz151*, *dz168* alleles indicated. Alignments of HST-6 with fly (*Dm*), mouse (*Mm*) and human (*Hs*) orthologs to show mutations within their molecular context. Asterisks and colons indicate perfectly conserved and well conserved residues, respectively. 3'PSB (phosphoadenosyl-phosphosulfate) binding site. SL1: the cDNA is transpliced to a SL1 leader sequence.

B. Scatterplot of chromosome V displaying the ratio of the number of sequencing reads showing the CB4856 (Hawaiian) single nucleotide polymorphism (SNP) at a given position divided by the total number of sequencing reads at that position for *dz165* using a combination of single nucleotide polymorphism and whole genome sequencing (DOITSIDOU *et al.* 2010). The mutations is to the left of the red line on the far end of chromosome V where this ratio approaches zero.

C. Schematic of the genomic and cDAN structure of the Xylosyltransferase *sqv-6* with the mutation indicated. TD: transmembrane domain, WSC: is a putative carbohydrate binding domain (present in yeast cell **w**all integrity and **s**tress response **c**omponent proteins). SL1: the cDNA is transpliced to a SL1 leader sequence.

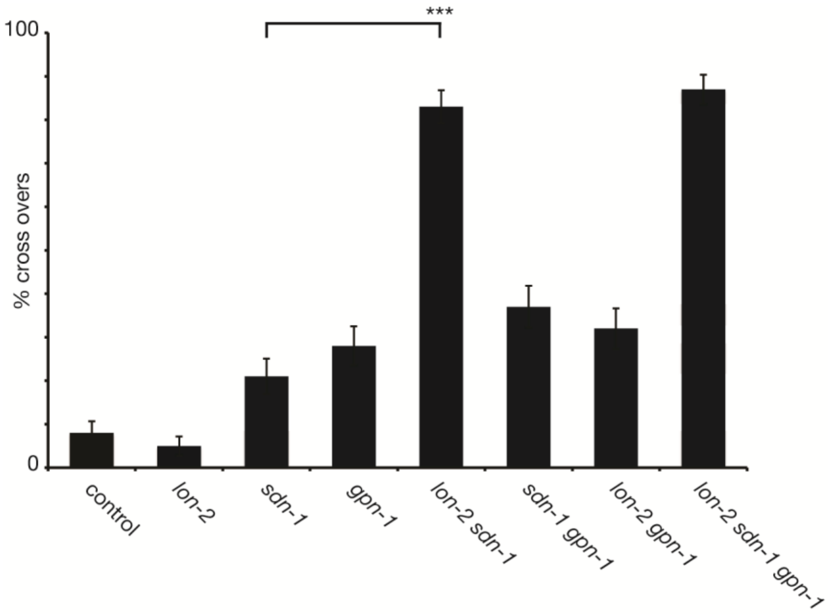

Figure S4 HSPG act redundantly to mediate midline patterning of the PVQ axons. Genetic analysis of PVQ midline patterning in HSPG mutant as as indicated. PVQ midline patterning was analyzed for crossing over phenotype. A significant enhancement was observed in the *lon-2(e678) sdn-1(zh20)* double mutant when compared to the single mutants. Asterisks denote statistical significance: ***p < 0.0005.

REFERENCES

Doitsidou, M., R. J. Poole, S. Sarin, H. Bigelow and O. Hobert, 2010 C. elegans mutant identification with a one-step whole-genome-sequencing and SNP mapping strategy. PLoS One 5**:** e15435.
