# Supplementary material for: Complex Cooperative Functions of Heparan Sulfate Proteoglycans Shape Nervous System Development in Caenorhabditis elegans
Source: G3 (Bethesda). 2014 Aug 5;4(10):1859–70. doi: 10.1534/g3.114.012591 (PMC4199693; doi:10.1534/g3.114.012591)
Supplement: Supporting Information [file supp_g3.114.012591_TableS1.pdf]

Table S1 Summary of genetic experiments for X-linkage and complementation

Data for mutants with cell body misplacement		
**Genotype**	**% misplacement**	**N**
dig-1(dz152)/+; *otIs76mgIs18/mgIs18; him-5(e1490)/+; otIs35/0 (♂)*	4	24
dig-1(dz136)/+; *otIs76mgIs18/mgIs18; him-5(e1490)/+; otIs35/0 (♂)*	0	15
dig-1(dz145)/+; *otIs76mgIs18/mgIs18; him-5(e1490)/+; otIs35/0 (♂)*	0	14
dig-1(dz154)/+; *otIs76mgIs18/mgIs18; him-5(e1490)/+; otIs35/0 (♂)*	0	32
dig-1(dz155)/+; *otIs76mgIs18/mgIs18; him-5(e1490)/+; otIs35/0 (♂)*	0	36
dig-1(dz152)/+; *otIs76mgIs18/mgIs18; him-5(e1490)/+; otIs35/+ (⚥)*	0	30
dig-1(dz136)/+; *otIs76mgIs18/mgIs18; him-5(e1490)/+; otIs35/+ (⚥)*	0	15
dig-1(dz145)/+; *otIs76mgIs18/mgIs18; him-5(e1490)/+; otIs35/+ (⚥)*	0	37
dig-1(dz154)/+; *otIs76mgIs18/mgIs18; him-5(e1490)/+; otIs35/+ (⚥)*	0	32
dig-1(dz155)/+; *otIs76mgIs18/mgIs18; him-5(e1490)/+; otIs35/+ (⚥)*	0	32
dig-1(dz152)/dig-1(ky388); *otIs76mgIs18/mgIs18; him-5(e1490)/+; otIs35/+ (⚥)*	76	50
dig-1(dz152)/dig-1(n1321); *otIs76mgIs18/mgIs18; him-5(e1490)/+; otIs35/+ (⚥)*	86	50
dig-1(dz136)/dig-1(dz152); *otIs76mgIs18/mgIs18; him-5(e1490)/+; otIs35/+ (⚥)*	16	75
dig-1(dz145)/dig-1(dz155); *otIs76mgIs18/mgIs18; him-5(e1490)/+; otIs35/+ (⚥)*	70	57
dig-1(dz154)/dig-1(dz152); *otIs76mgIs18/mgIs18; him-5(e1490)/+; otIs35/+ (⚥)*	92	36
dig-1(dz155)/dig-1(dz152); *otIs76mgIs18/mgIs18; him-5(e1490)/+; otIs35/+ (⚥)*	95	37
dig-1(dz152); *otIs76mgIs18; rhEx40 (Rescuing array) (⚥)*	5	100
**Data for mutants that suppress *kal-1*-dependent branching**	**% branching**	**N**
dig-1(dz136)/dig-1(dz152); *otIs76mgIs18/mgIs18; him-5(e1490)/+; otIs35/+ (⚥)*	69	75
otIs76mgIs18/mgIs18; him-5(e1490)/+; otIs35 ***hst-6(dz134)****/0 (♂)*	19	16
otIs76mgIs18/mgIs18; him-5(e1490)/+; otIs35 ***hst-6(dz151)****/0 (♂)*	10	30
otIs76mgIs18/mgIs18; him-5(e1490)/+; otIs35 ***hst-6(dz168)****/0 (♂)*	9	55
otIs76mgIs18/mgIs18; him-5(e1490)/+; otIs35 ***hst-6(dz134)****/+ (⚥)*	100	13
otIs76mgIs18/mgIs18; him-5(e1490)/+; otIs35 ***hst-6(dz151)****/+ (⚥)*	96	24
otIs76mgIs18/mgIs18; him-5(e1490)/+; otIs35 ***hst-6(dz168)****/+ (⚥)*	92	51
otIs76mgIs18/mgIs18; him-5(e1490)/+; ***+ hst-6(ok273)/otIs35 hst-6(dz134)*** *(⚥)*	4	50
otIs76mgIs18/mgIs18; him-5(e1490)/+; ***+ hst-6(ok273)/otIs35 hst-6(dz151)*** *(⚥)*	5	22
otIs76mgIs18/mgIs18; him-5(e1490)/+; ***+ hst-6(ok273)/otIs35 hst-6(dz168)*** *(⚥)*	6	50
otIs76mgIs18/mgIs18; him-5(e1490)/+; otIs35 ***hst-3.2(dz140)****/0 (♂)*	95	44
otIs76mgIs18/mgIs18; him-5(e1490)/+; otIs35 ***hst-3.2(dz140)****/+ (⚥)*	100	45
otIs76mgIs18/mgIs18; him-5(e1490)/+; ***+ hst-3.2(dz171)/otIs35 hst-3.2(dz140)*** *(⚥)*	12	50
otIs76mgIs18/mgIs18; ***sqv-6(dz165)****/+; otIs35/0 (♂)*	97	30
otIs76mgIs18/mgIs18; ***sqv-6(dz165)****/+; otIs35/+ (⚥)*	90	40
otIs76mgIs18/mgIs18; ***sqv-6(dz165)****/pst-1(ot20); otIs35/+ (⚥)*	20	20
otIs76mgIs18/mgIs18; ***sqv-6(dz165)****/hse-5(tm472); otIs35/+ (⚥)*	100	20
dz148/+; otIs76mgIs18/mgIs18; otIs35/0 (♂)	88	26
dz148/+; otIs76mgIs18/mgIs18; otIs35/+ (⚥)	91	34
**Data for mutants that enhance *kal-1*-dependent branching**	**% enhancement**	**N**
otIs76mgIs18/mgIs18; ***dz147****/+; otIs35/0 (♂)*	0	23
otIs76mgIs18/mgIs18; ***dz147****/+; otIs35/+ (⚥)*	0	24
otIs77/+; mgIs18/mgIs18; ***dz147/ot21****; otIs35/+ (⚥)*	56	50
