# Supplementary material for: Complex Cooperative Functions of Heparan Sulfate Proteoglycans Shape Nervous System Development in Caenorhabditis elegans
Source: G3 (Bethesda). 2014 Aug 5;4(10):1859–70. doi: 10.1534/g3.114.012591 (PMC4199693; doi:10.1534/g3.114.012591)
Supplement: Supporting Information [file supp_g3.114.012591_TableS2.pdf]

Table S2 List of Transgenic strains

Strain name	Constructs	Genotype
EB1543	*pttx-3::hst-6 and pmyo-3::mCherry*	*dzEx725; otIs76mgIs18IV; hst-6(ok273)X*
EB1544	*pttx-3::hst-6 and pmyo-3::mCherry*	*dzEx726; otIs76mgIs18IV; hst-6(ok273)X*
EB1691	*pttx-3::hst-6 and pmyo-3::mCherry*	*dzEx818; otIs76mgIs18IV; hst-6(ok273)X*
EB1692	*pttx-3::hst-6 and pmyo-3::mCherry*	*dzEx819; otIs76mgIs18IV; hst-6(ok273)X*
EB1693	*pttx-3::hst-6 and pmyo-3::mCherry*	*dzEx820; otIs76mgIs18IV; hst-6(ok273)X*
EB1698	*prgef-1::hst-6 and pmyo-3::mCherry*	*dzEx825; otIs76mgIs18IV; hst-6(ok273)X*
EB1699	*prgef-1::hst-6 and pmyo-3::mCherry*	*dzEx826; otIs76mgIs18IV; hst-6(ok273)X*
EB1489	*pdpy-7::hst-6 and pmyo-3::mCherry*	*dzEx685; otIs76mgIs18IV; hst-6(ok273)X*
EB1490	*pdpy-7::hst-6 and pmyo-3::mCherry*	*dzEx686; otIs76mgIs18IV; hst-6(ok273)X*
EB1491	*pdpy-7::hst-6 and pmyo-3::mCherry*	*dzEx687; otIs76mgIs18IV; hst-6(ok273)X*
EB2427	*pdpy-7::hst-6 and pmyo-3::mCherry*	*dzEx826; otIs76mgIs18IV; hst-6(ok273)X*
EB1492	*pdpy-7::hst-6 and pmyo-3::mCherry*	*dzEx688; otIs76mgIs18IV; hst-6(ok273)X*
EB1488	*pdpy-7::hst-6 and pmyo-3::mCherry*	*dzEx684; otIs76mgIs18IV; hst-6(ok273)X*
EB2428	*pdpy-7::hst-6 and pmyo-3::mCherry*	*dzEx826; otIs76mgIs18IV; hst-6(ok273)X*
EB1487	*pdpy-7::hst-6 and pmyo-3::mCherry*	*dzEx683; otIs76mgIs18IV; hst-6(ok273)X*
EB2429	*pmyo-3::hst-6 and pmyo-3::mCherry*	*dzEx683; otIs76mgIs18IV; hst-6(ok273)X*
EB1694	*pmyo-3::hst-6 and pmyo-3::mCherry*	*dzEx821; otIs76mgIs18IV; hst-6(ok273)X*
EB1486	*pmyo-3::hst-6 and pmyo-3::mCherry*	*dzEx682; otIs76mgIs18IV; hst-6(ok273)X*
EB2430	*pmyo-3::hst-6 and pmyo-3::mCherry*	*dzEx683; otIs76mgIs18IV; hst-6(ok273)X*
EB2431	*pmyo-3::hst-6 and pmyo-3::mCherry*	*dzEx683; otIs76mgIs18IV; hst-6(ok273)X*
EB1485	*pmyo-3::hst-6 and pmyo-3::mCherry*	*dzEx681; otIs76mgIs18IV; hst-6(ok273)X*
EB1700	*pdpy-7::hse-5 and pmyo-3::mCherry*	*dzEx827; hse-5(tm472)III; otIs76mgIs18IV*
EB1701	*pdpy-7::hse-5 and pmyo-3::mCherry*	*dzEx828; hse-5(tm472)III; otIs76mgIs18IV*
EB1702	*pdpy-7::hse-5 and pmyo-3::mCherry*	*dzEx829; hse-5(tm472)III; otIs76mgIs18IV*
EB1551	*pmyo-3::hse-5 and pmyo-3::mCherry*	*dzEx731; hse-5(tm472)III; otIs76mgIs18IV*
EB1789	*pmyo-3::hse-5 and pmyo-3::mCherry*	*dzEx883; hse-5(tm472)III; otIs76mgIs18IV*
EB1790	*pmyo-3::hse-5 and pmyo-3::mCherry*	*dzEx884; hse-5(tm472)III; otIs76mgIs18IV*
EB1791	*pmyo-3::hse-5 and pmyo-3::mCherry*	*dzEx885; hse-5(tm472)III; otIs76mgIs18IV*
EB1792	*pmyo-3::hse-5 and pmyo-3::mCherry*	*dzEx886; hse-5(tm472)III; otIs76mgIs18IV*
EB1793	*pmyo-3::hse-5 and pmyo-3::mCherry*	*dzEx887; hse-5(tm472)III; otIs76mgIs18IV*
EB1730	*pttx-3::hst-2 and pmyo-3::mCherry*	*dzEx838; otIs76mgIs18IV; hst-2(ok595)X*
EB1731	*pttx-3::hst-2 and pmyo-3::mCherry*	*dzEx839; otIs76mgIs18IV; hst-2(ok595)X*
EB1732	*pttx-3::hst-2 and pmyo-3::mCherry*	*dzEx840; otIs76mgIs18IV; hst-2(ok595)X*
EB1733	*pttx-3::hst-2 and pmyo-3::mCherry*	*dzEx841; otIs76mgIs18IV; hst-2(ok595)X*
EB1734	*pttx-3::hst-2 and pmyo-3::mCherry*	*dzEx842; otIs76mgIs18IV; hst-2(ok595)X*
EB1735	*pttx-3::hst-2 and pmyo-3::mCherry*	*dzEx843; otIs76mgIs18IV; hst-2(ok595)X*
EB1736	*pttx-3::hst-2 and pmyo-3::mCherry*	*dzEx844; otIs76mgIs18IV; hst-2(ok595)X*
EB1737	*pttx-3::hst-2 and pmyo-3::mCherry*	*dzEx845; otIs76mgIs18IV; hst-2(ok595)X*
EB1738	*pttx-3::hst-2 and pmyo-3::mCherry*	*dzEx846; otIs76mgIs18IV; hst-2(ok595)X*
EB1739	*pttx-3::hst-2 and pmyo-3::mCherry*	*dzEx847; otIs76mgIs18IV; hst-2(ok595)X*
EB1742	*prgef-1::hst-2 and pmyo-3::mCherry*	*dzEx848; otIs76mgIs18IV; hst-2(ok595)X*
EB1743	*prgef-1::hst-2 and pmyo-3::mCherry*	*dzEx849; otIs76mgIs18IV; hst-2(ok595)X*
EB1744	*prgef-1::hst-2 and pmyo-3::mCherry*	*dzEx850; otIs76mgIs18IV; hst-2(ok595)X*
EB1745	*prgef-1::hst-2 and pmyo-3::mCherry*	*dzEx851; otIs76mgIs18IV; hst-2(ok595)X*
EB1749	*pdpy-7::hst-2 and pmyo-3::mCherry*	*dzEx854; otIs76mgIs18IV; hst-2(ok595)X*
EB1818	*pdpy-7::hst-2 and pmyo-3::mCherry*	*dzEx912; otIs76mgIs18IV; hst-2(ok595)X*
EB1819	*pdpy-7::hst-2 and pmyo-3::mCherry*	*dzEx913; otIs76mgIs18IV; hst-2(ok595)X*
EB1747	*pmyo-3::hst-2 and pmyo-3::mCherry*	*dzEx852; otIs76mgIs18IV; hst-2(ok595)X*
EB1748	*pmyo-3::hst-2 and pmyo-3::mCherry*	*dzEx853; otIs76mgIs18IV; hst-2(ok595)X*
EB1823	*pmyo-3::hst-2 and pmyo-3::mCherry*	*dzEx915; otIs76mgIs18IV; hst-2(ok595)X*
EB1756	*pttx-3::hse-5 and pmyo-3::mCherry*	*dzEx858; hse-5(tm472)III; otIs76mgIs18IV*
EB1757	*pttx-3::hse-5 and pmyo-3::mCherry*	*dzEx859; hse-5(tm472)III; otIs76mgIs18IV*
EB1758	*pttx-3::hse-5 and pmyo-3::mCherry*	*dzEx860; hse-5(tm472)III; otIs76mgIs18IV*
EB1750	*prgef-1::hse-5 and pmyo-3::mCherry*	*dzEx855; hse-5(tm472)III; otIs76mgIs18IV*
EB1754	*prgef-1::hse-5 and pmyo-3::mCherry*	*dzEx856; hse-5(tm472)III; otIs76mgIs18IV*

EB1755	*prgef-1::hse-5 and pmyo-3::mCherry*	*dzEx857; hse-5(tm472)III; otIs76mgIs18IV*
EB1786	*prgef-1::hse-5 and pmyo-3::mCherry*	*dzEx880; hse-5(tm472)III; otIs76mgIs18IV*
EB1787	*prgef-1::hse-5 and pmyo-3::mCherry*	*dzEx881; hse-5(tm472)III; otIs76mgIs18IV*
EB1788	*prgef-1::hse-5 and pmyo-3::mCherry*	*dzEx882; hse-5(tm472)III; otIs76mgIs18IV*
EB2396	*prgef-1::sqv-6 and pmyo-3::mCherry*	*dzEx1337; otIs76mgIs18IV; sqv-6(dz165)X*
EB2397	*prgef-1::sqv-6 and pmyo-3::mCherry*	*dzEx1338; otIs76mgIs18IV; sqv-6(dz165)X*
EB2398	*prgef-1::sqv-6 and pmyo-3::mCherry*	*dzEx1339; otIs76mgIs18IV; sqv-6(dz165)X*
EB2399	*prgef-1::sqv-6 and pmyo-3::mCherry*	*dzEx1340; otIs76mgIs18IV; sqv-6(dz165)X*
EB2400	*pdpy-7::sqv-6 and pmyo-3::mCherry*	*dzEx1341; otIs76mgIs18IV; sqv-6(dz165)X*
EB2401	*pdpy-7::sqv-6 and pmyo-3::mCherry*	*dzEx1342; otIs76mgIs18IV; sqv-6(dz165)X*
EB2402	*pdpy-7::sqv-6 and pmyo-3::mCherry*	*dzEx1343; otIs76mgIs18IV; sqv-6(dz165)X*
EB2403	*pdpy-7::sqv-6 and pmyo-3::mCherry*	*dzEx1344; otIs76mgIs18IV; sqv-6(dz165)X*
EB2404	*pdpy-7::sqv-6 and pmyo-3::mCherry*	*dzEx1345; otIs76mgIs18IV; sqv-6(dz165)X*
EB2405	*pdpy-7::sqv-6 and pmyo-3::mCherry*	*dzEx1346; otIs76mgIs18IV; sqv-6(dz165)X*
EB2406	*pmyo-3::sqv-6 and pmyo-3::mCherry*	*dzEx1347; otIs76mgIs18IV; sqv-6(dz165)X*
EB2407	*pmyo-3::sqv-6 and pmyo-3::mCherry*	*dzEx1348; otIs76mgIs18IV; sqv-6(dz165)X*
EB2408	*pmyo-3::sqv-6 and pmyo-3::mCherry*	*dzEx1349; otIs76mgIs18IV; sqv-6(dz165)X*
EB2409	*pmyo-3::sqv-6 and pmyo-3::mCherry*	*dzEx1350; otIs76mgIs18IV; sqv-6(dz165)X*
EB2410	*pmyo-3::sqv-6 and pmyo-3::mCherry*	*dzEx1351; otIs76mgIs18IV; sqv-6(dz165)X*
EB2411	*pmyo-3::sqv-6 and pmyo-3::mCherry*	*dzEx1352; otIs76mgIs18IV; sqv-6(dz165)X*
EB2412	*pmyo-3::sqv-6 and pmyo-3::mCherry*	*dzEx1353; otIs76mgIs18IV; sqv-6(dz165)X*
EB2413	*pmyo-3::sqv-6 and pmyo-3::mCherry*	*dzEx1354; otIs76mgIs18IV; sqv-6(dz165)X*
EB2414	*pmyo-3::sqv-6 and pmyo-3::mCherry*	*dzEx1355; otIs76mgIs18IV; sqv-6(dz165)X*
EB2415	*pmyo-3::sqv-6 and pmyo-3::mCherry*	*dzEx1356; otIs76mgIs18IV; sqv-6(dz165)X*
EB2416	*pmyo-3::sqv-6 and pmyo-3::mCherry*	*dzEx1357; otIs76mgIs18IV; sqv-6(dz165)X*
EB2417	*pttx-3::sqv-6 and pmyo-3::mCherry*	*dzEx1358; otIs76mgIs18IV; sqv-6(dz165)X*
EB2418	*pttx-3::sqv-6 and pmyo-3::mCherry*	*dzEx1359; otIs76mgIs18IV; sqv-6(dz165)X*
EB2419	*pttx-3::sqv-6 and pmyo-3::mCherry*	*dzEx1360; otIs76mgIs18IV; sqv-6(dz165)X*
