# Supplementary material for: Complex Cooperative Functions of Heparan Sulfate Proteoglycans Shape Nervous System Development in Caenorhabditis elegans
Source: G3 (Bethesda). 2014 Aug 5;4(10):1859–70. doi: 10.1534/g3.114.012591 (PMC4199693; doi:10.1534/g3.114.012591)
Supplement: Supporting Information [file supp_g3.114.012591_FigureS1.pdf]

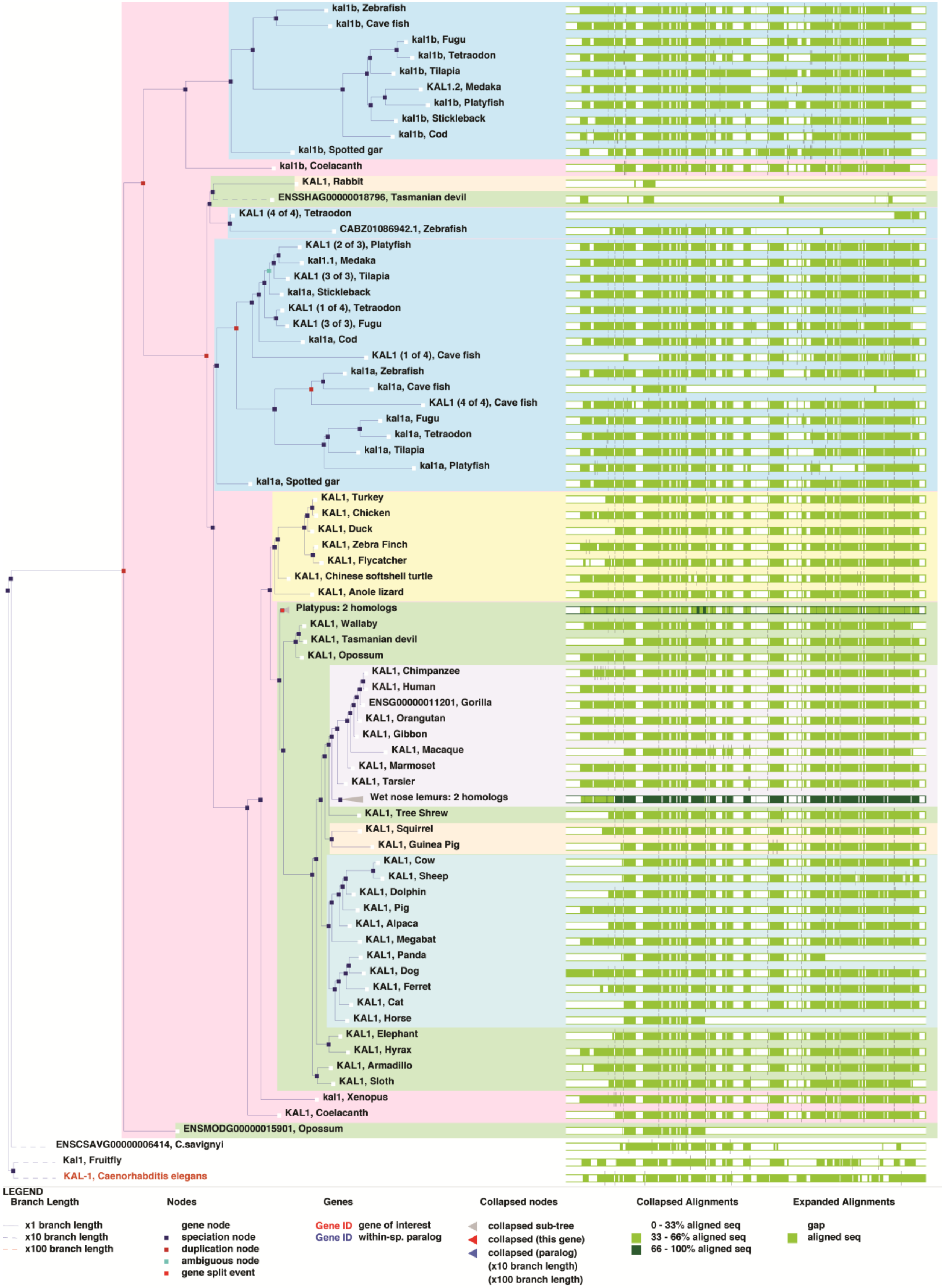
kal1b, Zebrafish
kal1b, Cave fish
kal1b, Fugu
kal1b, Tetraodon
kal1b, Tilapia
KAL1.2, Medaka
kal1b, Platyfish
kal1b, Stickleback
kal1b, Cod
kal1b, Spotted gar
kal1b, Coelacanth
KAL1, Rabbit
ENSSHAG00000018796, Tasmanian devil
KAL1 (4 of 4), Tetraodon
CABZ01086942.1, Zebrafish
KAL1 (2 of 3), Platyfish
kal1.1, Medaka
KAL1 (3 of 3), Tilapia
kal1a, Stickleback
KAL1 (1 of 4), Tetraodon
KAL1 (3 of 3), Fugu
kal1a, Cod
KAL1 (1 of 4), Cave fish
kal1a, Zebrafish
kal1a, Cave fish
KAL1 (4 of 4), Cave fish
kal1a, Fugu
kal1a, Tetraodon
kal1a, Tilapia
kal1a, Platyfish
kal1a, Spotted gar
KAL1, Turkey
KAL1, Chicken
KAL1, Duck
KAL1, Zebra Finch
KAL1, Flycatcher
KAL1, Chinese softshell turtle
KAL1, Anole lizard
Platypus: 2 homologs
KAL1, Wallaby
KAL1, Tasmanian devil
KAL1, Opossum
KAL1, Chimpanzee
KAL1, Human
ENSG00000011201, Gorilla
KAL1, Orangutan
KAL1, Gibbon
KAL1, Macaque
KAL1, Marmoset
KAL1, Tarsier
Wet nose lemurs: 2 homologs
KAL1, Tree Shrew
KAL1, Squirrel
KAL1, Guinea Pig
KAL1, Cow
KAL1, Sheep
KAL1, Dolphin
KAL1, Pig
KAL1, Alpaca
KAL1, Megabat
KAL1, Panda
KAL1, Dog
KAL1, Ferret
KAL1, Cat
KAL1, Horse
KAL1, Elephant
KAL1, Hyrax
KAL1, Armadillo
KAL1, Sloth
kal1, Xenopus
KAL1, Coelacanth
ENSMODG00000015901, Opossum
ENSCSAVG00000006414, C.savignyi
Kal1, Fruitfly
KAL-1, Caenorhabditis elegans
LEGEND
Branch Length
x1 branch length
x10 branch length
x100 branch length
Nodes
gene node
speciation node
duplication node
ambiguous node
gene split event
Genes
Gene ID gene of interest
Gene ID within-sp. paralog
Collapsed nodes
collapsed sub-tree
collapsed (this gene)
collapsed (paralog)
(x10 branch length)
(x100 branch length)
Collapsed Alignments
0 - 33% aligned seq
33 - 66% aligned seq
66 - 100% aligned seq
Expanded Alignments
gap
aligned seq

Figure S1 Phylogenetic Tree of KAL1/anosmin-1 proteins. Phylogenetic tree of KAL1/anosmin-1 obtained from http://www.ensembl.org. Accession number for this tree is ENSGT00440000033720. *C. elegans* KAL-1 is marked in red.
