# Supplementary material for: Complex Cooperative Functions of Heparan Sulfate Proteoglycans Shape Nervous System Development in Caenorhabditis elegans
Source: G3 (Bethesda). 2014 Aug 5;4(10):1859–70. doi: 10.1534/g3.114.012591 (PMC4199693; doi:10.1534/g3.114.012591)
Supplement: Supporting Information [file supp_g3.114.012591_FigureS2.pdf]

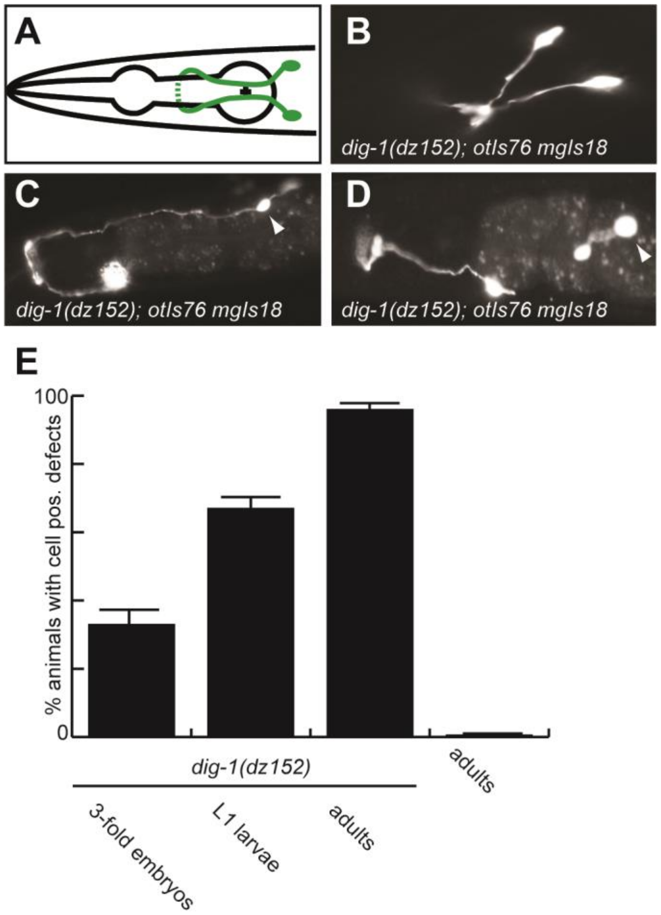

Figure S2 ***dig-1*** **cell positioning defects in AIY.**

A-D: Representative images of *dig-1* defects in AIY. Diagram of a ventral view of AIY in an adult wild type animal (A), *dig-1* mediated suppression of the *kal-1*-dependent branching in AIY (B), *dig-1* suppression of the *kal-1*-dependent branching in AIY and mispositioning of the AIY cell body (C), and mispositioning of the AIY cell body in a *dig-1* mutant independent of the *kal-1 gof* branching in AIY (D). Arrowheads indicate mispositioned AIY cell bodies.

E. Quantification of animals with cell positioning defects at the developmental stages and genotypes indicated All experiments were performed in a AIY *otIs76 mgIs18(Is[Pttx-3::kal-1, Pttx-3::gfp]* background. Error bars indicate the standard error of proportion. N≥100 in all cases.
