# Supplementary material for: Complex Cooperative Functions of Heparan Sulfate Proteoglycans Shape Nervous System Development in Caenorhabditis elegans
Source: G3 (Bethesda). 2014 Aug 5;4(10):1859–70. doi: 10.1534/g3.114.012591 (PMC4199693; doi:10.1534/g3.114.012591)
Supplement: Supporting Information [file supp_g3.114.012591_FigureS4.pdf]

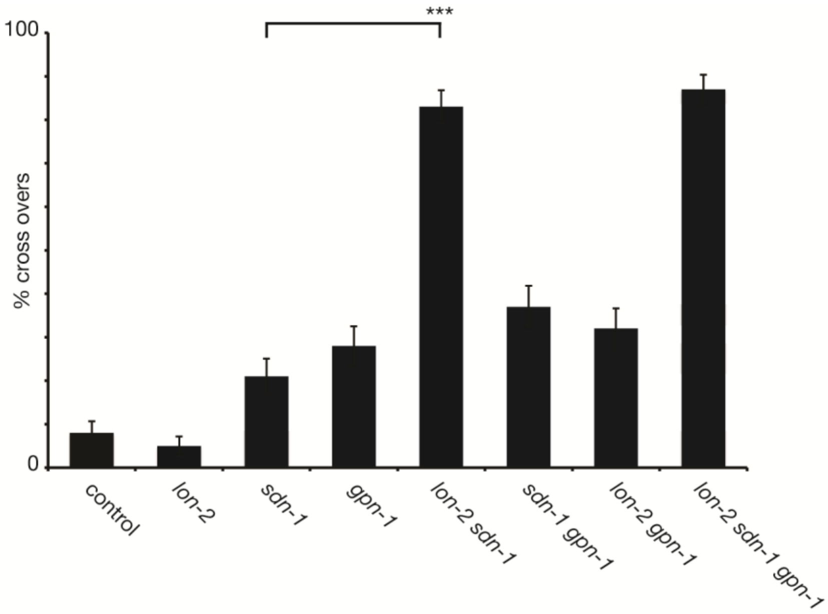

Figure S4 HSPG act redundantly to mediate midline patterning of the PVQ axons. Genetic analysis of PVQ midline patterning in HSPG mutant as as indicated. PVQ midline patterning was analyzed for crossing over phenotype. A significant enhancement was observed in the *lon-2(e678) sdn-1(zh20)* double mutant when compared to the single mutants. Asterisks denote statistical significance: ***p < 0.0005.

REFERENCES

Doitsidou, M., R. J. Poole, S. Sarin, H. Bigelow and O. Hobert, 2010 C. elegans mutant identification with a one-step whole-genome-sequencing and SNP mapping strategy. PLoS One 5**:** e15435.
