# Supplementary material for: Complex Cooperative Functions of Heparan Sulfate Proteoglycans Shape Nervous System Development in Caenorhabditis elegans
Source: G3 (Bethesda). 2014 Aug 5;4(10):1859–70. doi: 10.1534/g3.114.012591 (PMC4199693; doi:10.1534/g3.114.012591)
Supplement: Supporting Information [file supp_g3.114.012591_FigureS3.pdf]

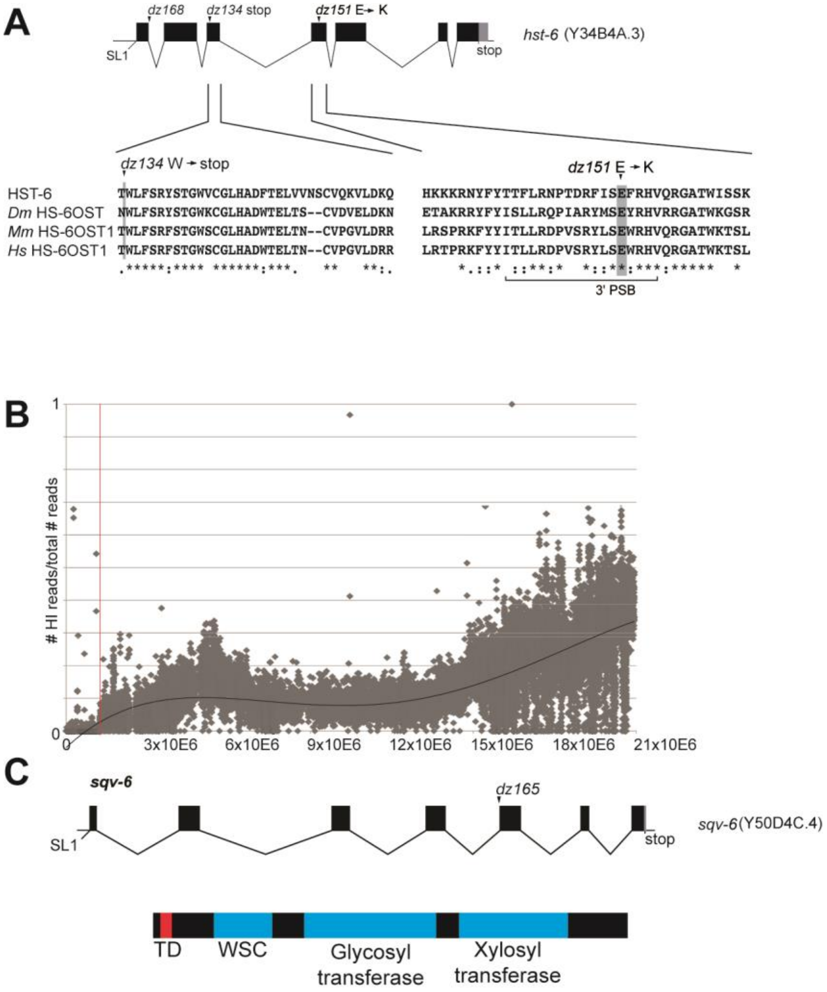

Figure S3 Molecular lesions in *hst-6* and *sqv-6.*

A. *hst-6* gene structure with molecular lesions of *dz134*, and *dz151*, *dz168* alleles indicated. Alignments of HST-6 with fly (*Dm*), mouse (*Mm*) and human (*Hs*) orthologs to show mutations within their molecular context. Asterisks and colons indicate perfectly conserved and well conserved residues, respectively. 3'PSB (phosphoadenosyl-phosphosulfate) binding site. SL1: the cDNA is transpliced to a SL1 leader sequence.

B. Scatterplot of chromosome V displaying the ratio of the number of sequencing reads showing the CB4856 (Hawaiian) single nucleotide polymorphism (SNP) at a given position divided by the total number of sequencing reads at that position for *dz165* using a combination of single nucleotide polymorphism and whole genome sequencing (DOITSIDOU *et al.* 2010). The mutations is to the left of the red line on the far end of chromosome V where this ratio approaches zero.

C. Schematic of the genomic and cDAN structure of the Xylosyltransferase *sqv-6* with the mutation indicated. TD: transmembrane domain, WSC: is a putative carbohydrate binding domain (present in yeast cell **w**all integrity and **s**tress response **c**omponent proteins). SL1: the cDNA is transpliced to a SL1 leader sequence.
